# Analysis of the complete plastomes and nuclear ribosomal DNAs from *Euonymus hamiltonianus* and its relatives sheds light on their diversity and evolution

**DOI:** 10.1371/journal.pone.0275590

**Published:** 2022-10-05

**Authors:** Young Sang Park, Jong-Soo Kang, Jee Young Park, Hyeonah Shim, Hyun Ok Yang, Jung Hwa Kang, Tae-Jin Yang

**Affiliations:** 1 Department of Agriculture, Forestry and Bioresources, Plant Genomics & Breeding Institute, College of Agriculture & Life Sciences, Seoul National University, Seoul, Korea; 2 Department of Integrative Biological Sciences and Industry, Sejong University, Seoul, Korea; 3 Hantaek Botanical Garden, Yongin, Korea; Midwestern University, UNITED STATES

## Abstract

*Euonymus hamiltonianus* and its relatives (Celastraceae family) are used for ornamental and medicinal purposes. However, species identification in *Euonymus* is difficult due to their morphological diversity. Using plastid genome (plastome) data, we attempt to reveal phylogenetic relationship among *Euonymus* species and develop useful markers for molecular identification. We assembled the plastome and nuclear ribosomal DNA (nrDNA) sequences from five *Euonymus* lines collected from South Korea: three *Euonymus hamiltonianus* accessions, *E*. *europaeus*, and *E*. *japonicus*. We conducted an in-depth comparative analysis using ten plastomes, including other publicly available plastome data for this genus. The genome structures, gene contents, and gene orders were similar in all *Euonymus* plastomes in this study. Analysis of nucleotide diversity revealed six divergence hotspots in their plastomes. We identified 339 single nucleotide polymorphisms and 293 insertion or deletions among the four *E*. *hamiltonianus* plastomes, pointing to abundant diversity even within the same species. Among 77 commonly shared genes, 9 and 33 were identified as conserved genes in the genus *Euonymus* and *E*. *hamiltonianus*, respectively. Phylogenetic analysis based on plastome and nrDNA sequences revealed the overall consensus and relationships between plastomes and nrDNAs. Finally, we developed six barcoding markers and successfully applied them to 31 *E*. *hamiltonianus* lines collected from South Korea. Our findings provide the molecular basis for the classification and molecular taxonomic criteria for the genus *Euonymus* (at least in Korea), which should aid in more objective classification within this genus. Moreover, the newly developed markers will be useful for understanding the species delimitation of *E*. *hamiltonianus* and closely related species.

## Introduction

*Euonymus hamiltonianus* Wall., a plant belonging to the Celastraceae family, is widely distributed from Northern India to Far East Asia (http://www.plantsoftheworldonline.org). *E*. *hamiltonianus* are valuable ornamental plants due to their beautiful shapes and colors. However, the morphological variations lead to ambiguous delimitation of this species. The uncertainty of species delimitation is not only the case for *E*. *hamiltonianus* but also for other closely related species. Fruit morphology of *Euonymus* was used to divide them into five sections, but molecular phylogeny of *Euonymus* was poorly supported in the previous study [1].

Although Celastraceae is a large family containing 96 genera and 1,350 species, few studies have been conducted for the genus *Euonymus* [2–4]. Universal DNA barcoding regions such as plastid *matK*, *rbcL*, and nuclear ribosomal internal transcribed spacer (nrITS) regions have been utilized to construct the phylogenies of Celastraceae [5–8]. Nevertheless, the intrageneric boundaries are still unclear due to the limitations of using a few short barcoding regions. To date, most studies in *Euonymus* have been conducted on individual species [9–12]. Even though an interspecies comparison of the *Euonymus* plastomes was recently conducted, no in-depth analysis of one species with its relatives has been performed [3]. Therefore, to address the plastome diversity and evolution of *E*. *hamiltonianus* and its relatives, it is important to conduct in-depth comparative analysis using various resources.

Plastomes have been widely employed for phylogenetic studies and DNA barcoding in various plants due to their uniparental inheritance (usually maternal inheritance) in most land plants [13]. Uniparental inheritance could reduce genetic diversity, but also provide simplicities in tracking ancestors (usually maternal) and obtaining genetic information with less heterogeneity [14]. In most land plants, plastid and mitochondrial genomes exhibit contrasting patterns in genomic features such as genome size, genome structure, gene content, and nucleotide substitution rates. Although plastomes have conserved genome structure and gene content, nucleotide substitution rates of plastid genes are generally faster than those of mitochondrial genes. In contrast, mitochondrial genomes display complicated genome structure and variable gene content, but substitution rates of mitochondrial genes are slower than those of plastid genes [15–18]. These genomic features have allowed plastomes to accumulate variations among species at a moderate rate [19,20].

Nuclear ribosomal DNAs such as 45S rDNA and 5S rDNA exist in a tandem repeat array of hundreds to thousands of copies and are genetically conserved due to concerted evolution [21,22]. However, the ITS regions, ITS1 between 18S and 5.8S and ITS2 between 5.8S and 26S, of the 45S rDNA are relatively variable at the sequence level. These characteristics of the nrDNA region provide useful genetic information for phylogenetic studies and DNA barcoding [23]. In addition, due to the development of Next Generations Sequencing (NGS) techniques, the assembly of complete plastome and 45S rDNA sequences can be performed quickly in a cost-effective manner. Complete plastome and 45S rDNA data generated by NGS platforms are quite helpful for species identification [24–29].

In this study, we assembled the complete plastomes and nrDNA (45S and 5S) sequences of five *Euonymus* plants by *de novo* assembly of low-coverage whole-genome shotgun sequencing (dnaLCW) [27,28]. We also conducted an in-depth analysis of the genetic features of other *Euonymus* plastomes using data from NCBI GenBank (https://www.ncbi.nlm.nih.gov/genbank/). The newly discovered genetic features of *E*. *hamiltonianus* and its relatives advance our understanding of the molecular identification and plastome evolution of these species.

## Materials and methods

### Plant materials and genome sequencing

Thirty-one *E*. *hamiltonianus*, one *E*. *europaeus*, and one *E*. *japonicus* were collected from various sources including wild and commercial samples (S6 Table). The collected samples from Hantaek Botanical Garden (Baegam, Cheoin, Yongin, Gyeonggi, Korea) and Sannae Botanical Garden (Byeongcheon, Dongnam, Cheonan, South Chungcheon, Korea) were collected with permission from garden authorities (Permitted by Taek Joo Lee and Myung Hyoe Kim, president of Hantaek Botanical Garden and Sannae Botanical Garden, respectively). For samples collected from the wild, no specific permission was required for collecting the species in this study, according to the national and local legislations. Leaf samples were collected, flash frozen, and used for genomic DNA extraction using an Exgene Plant SV Midi Kit and an Exgene Plant SV Mini Kit (Geneall Biotechnology, Seoul) following the manufacturer’s protocols. The concentration and quality of the extracted genomic DNA were examined by gel electrophoresis and with a Nanodrop 2000 spectrometer (Thermo Scientific, USA). Among all 33 accessions, three *E*. *hamiltonianus*, one *E*. *europaeus* and one *E*. *japonicus* were used to generate paired-end (PE) libraries. The libraries were sequenced using the Illumina MiSeq platform at Phyzen (www.phyzen.com, Seongnam, Republic of Korea). This analysis yielded 0.82–1.20 Gbp of sequencing data per sample.

### Plastome and nrDNA assembly

All newly assembled plastomes and 45S rDNA were assembled using the dnaLCW method [27,28]. In summary, raw reads were trimmed by the trimming tool embedded in the CLC assembly cell. Trimmed reads were assembled into contigs by *de novo* assembly using the CLC assembly cell (ver.4.21, CLC Bio, Denmark). Among assembled contigs, only contigs with similarity to the reference plastome (*E*. *hamiltonianus*, NC_037518.1) were extracted by MUMmer and BLASTZ [30,31]. Finally, the plastome sequences were completed by manual curation. The completed plastomes were annotated using GeSeq (https://chlorobox.mpimp-golm.mpg.de/geseq) and Artemis [32,33].

Assembly of 45S rDNAs was performed in a similar manner following the pipeline used for plastome assembly. Only contigs with sequence similarity to the reference 45S rDNA (*E*. *hamiltonianus*, KY921875.1) were extracted. The start and end position of each 45S rDNA subunit (18S, ITS1, 5.8S, ITS2, and 26S) were determined using RNAmmer and the reference sequence [34].

Assembly of 5S rDNA was performed by reference mapping. Briefly, the draft sequence of 5S rDNA was assembled by mapping raw reads to the reference sequence of *Arabidopsis thaliana* (AF330993.1). After completing the assembly of 5S rDNA, the IGS region was assembled by mapping and elongation. Elongation of the IGS region was repeated until the mapped reads met the next 5S rDNA unit. Finally, the complete 5S rDNA unit with IGS sequences was confirmed via NCBI BLAST [35].

### Comparative analysis of plastomes

In addition to the five newly assembled plastomes, five other previously reported *Euonymus* plastome sequences were obtained from NCBI GenBank (Table 1). The similarity among plastomes was confirmed by the mVISTA with LAGAN alignment method using *E*. *hamiltonianus* (NC_037518.1) as a reference sequence with default parameters [36,37]. Structural variations between junction regions were identified by IRscope (https://irscope.shinyapps.io/irapp/) [38].

**Table 1:** Information about the plastomes of members of the *Euonymus* genus examined in this study.

Species	Length (bp)				Number of Genes			GenBank accession no.
	Total	LSC	IR	SSC	CDS	tRNA	rRNA	
*E*. *hamiltonianus* (Hantaek)	157,360	86,399	26,322	18,317	87	37	8	NC_037518.1
*E*. *hamiltonianus* (Hongcheon)	157,456	86,481	26,330	18,315	87	37	8	MZ567069*
*E*. *hamiltonianus* (Jeju)	157,511	86,518	26,339	18,315	87	37	8	MZ567070*
*E*. *hamiltonianus* (’Snow’)	157,536	86,532	26,340	18,324	87	37	8	MZ567071*
*E*. *europaeus*	157,263	86,245	26,344	18,330	87	37	8	MZ567072*
*E*. *japonicus*	157,628	85,909	26,700	18,319	88	37	8	MZ567073*
*E*. *japonicus*	157,637	85,903	26,697	18,340	88	37	8	NC_028067.1
*E*. *fortunei*	157,639	85,855	26,719	18,346	88	37	8	MH150885.1
*E*. *schensianus*	157,702	86,026	26,484	18,708	88	37	8	NC_036019.1
*E*. *szechuanensis*	157,465	86,257	26,368	18,472	87	37	8	NC_047463.1

LSC: Large Single Copy, IR: Inverted Repeat, SSC: Small Single Copy.

*: Newly assembled in this study.

To check the variants in whole plastomes, the ten *Euonymus* plastomes were aligned using the PRANK aligner with the +F option [39–41]. The aligned sequences were used to draw a plastome map and calculate the nucleotide diversity (pi value). Plastome gene and variation maps were drawn with circos-0.69–9 (http://circos.ca/) [42]. Pi values were calculated to estimate divergence hotspots in the whole plastome using DnaSP v6 by the sliding window method (window size: 600 bp, sliding size: 200 bp) [43].

### Comparative analysis of protein-coding gene sequences

Ten *Euonymus* plastomes and one *Catha edulis* plastome (GenBank accession No.: KT861471) were used for comparative analysis. Among the 88 protein-coding genes, the sequences of 77 genes were collected because they were nonredundant and shared by the 11 individuals. The dN/dS analysis was performed on these common genes using a branch-site model (model: 2, NSsites: 2) of codeml in the paml version 3.14 package [44]. A likelihood-ratio test (LRT) was performed on the analyzed values to identify candidate genes (df = 1, p-value < 0.05). Only genes with > 0.7 posterior probability were selected as putative selected genes among the candidate genes.

### Phylogenetic analysis

The 77 protein-coding gene sequences were independently aligned using PRANK aligner with the +F and translate option, and then were concatenated as a single supermatrix [39,40]. The concatenated supermatrix was used to reconstruct a plastome-based phylogenetic tree. The best substitution model for the supermatrix was selected by jModelTest version 2.1.10 via Akaike Information Criterion (AIC) analysis [45]. As a result, the GTR+Γ+I model was selected to be the best-fitting model. Based on the model test result, a Bayesian Inference (BI) tree was constructed using MrBayes v. 3.2.7 (rates = invgamma, ngen = 1,000,000, burninfrac = 0.25), while a Maximum Likelihood (ML) tree was constructed using RAxML GUI 2.0 with a rapid bootstrap test with 1,000 replicates and GTRGAMMAI [46,47].

To reconstruct the 45S rDNA phylogeny, species in Table 2 were aligned with MAFFT web version (https://mafft.cbrc.jp/alignment/software/) [48]. The substitution model was tested using the alignment sequences with jModelTest version 2.1.10 by AIC analysis [45]. Similar to the analysis with plastomes, GTR+Γ+I model was selected to be the best substitution model. With the selected model, ML and BI trees of 45S rDNA were constructed with the same condition for analyzing plastomes by RAxML GUI2.0 and MrBayes, respectively [46,47].

**Table 2:** Information about the nuclear ribosomal DNA (nrDNA) assembly of the six *Euonymus* accessions.

	Length (bp)							GenBank accession no.
Regions	45S rDNA					5S rDNA		
Species	18S RNA	ITS1	5.8S RNA	ITS2	26S RNA	5S RNA	IGS	
*E*. *hamiltonianus* (Hantaek)	1,809	237	164	216	3,398	121	343	KY921875.1/MZ556116*
*E*. *hamiltonianus* (Hongcheon)	1,809	237	164	215	3,398	121	343	MZ520609*/MZ556115*
*E*. *hamiltonianus* (Jeju)	1,809	237	164	216	3,398	121	343	MZ520610*/MZ556116*
*E*. *hamiltonianus* (’Snow’)	1,809	237	164	218	3,397	121	344	MZ520611*/MZ556117*
*E*. *europaeus*	1,809	237	164	216	3,397	121	347	MZ520612*/MZ556118*
*E*. *japonicus*	1,809	231	164	219	3,399	121	139	MZ520613*/MZ556119*

GenBank accession numbers for 45S (top) and 5S (bottom).

*: Newly assembled in this study.

### Marker development and validation

Three single nucleotide polymorphisms (SNPs) and three insertion or deletions (InDels) markers were developed based on the plastome variations among four *E*. *hamiltonianus* individuals. Three SNP markers were developed into dominant markers, while three InDel markers were codominant. All marker sets were subjected to *in silico* validation with NCBI Primer-BLAST (https://www.ncbi.nlm.nih.gov/tools/primer-blast/) [49]. The newly developed markers were validated through gel-based analyses with 32 *Euonymus* samples. The following were the PCR amplification conditions: Initial denaturation: 94°C for 5 minutes, denaturation: 94°C for 20 seconds, annealing: 58°C for 20 seconds, extension: 72°C for 20 seconds, final extensions: 72°C for 7 minutes. Denaturation, annealing and extension were conducted 35 cycles. After that, the PCR amplicon was validated by 3% agarose gel with gel-electrophoresis. *E*. *japonicus* (section *Ilicifolii*) was excluded from this validation, because the species was phylogenetically distant from *E*. *hamiltonianus* and *E*. *europaeus* (sect. *Euonymus*). Grouping of each accession was conducted with PowerMarker v3.25 by the NJ method with 1,000 bootstrap replicates [50]. Finally, the consensus tree was retrieved by consensus with the phylip package version 3.697 [51].

## Results

### Characteristics of plastomes and nrDNA

We assembled complete plastomes, 45S nrDNA, and 5S nrDNA sequences from five *Euonymus* lines. By adding plastome sequences from NCBI, a total of ten complete plastomes, six 45S rDNA sequences, and six 5S rDNA sequences were compared in downstream analyses.

**Fig 1:**
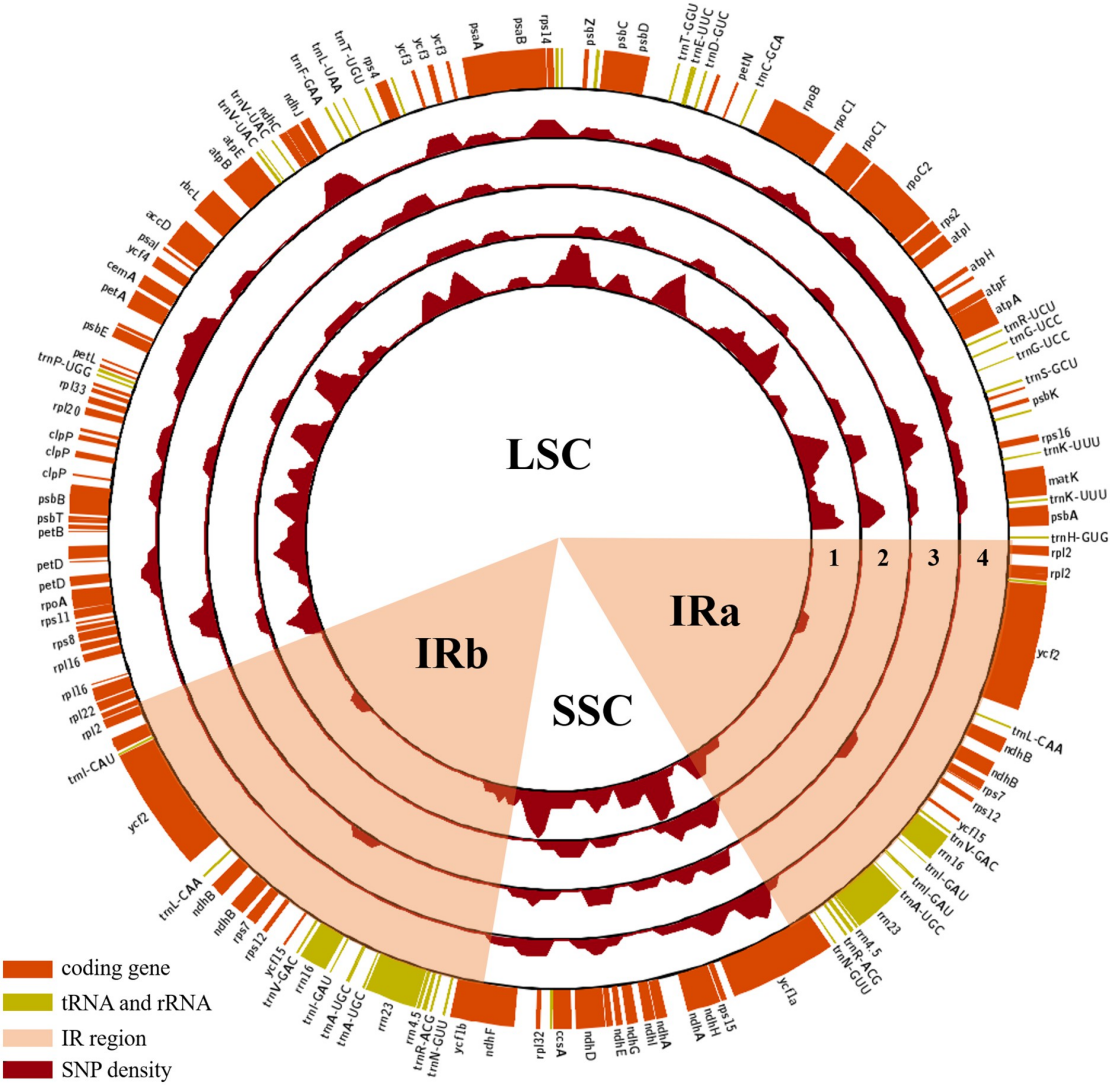
Gene map and plastome variations. SNPs were measured in every 2 kb sliding window with a 500 bp sliding length. 1–4: Maximum variant number scale in each map is 6; 1. *E*. *hamiltonianus* (Hantaek)- specific SNP density map; 2. *E*. *hamiltonianus* (Hongcheon)-specific SNP density map; 3. *E*. *hamiltonianus* (Jeju)-specific SNP density map; 4. *E*. *hamiltonianus* (‘Snow’)-specific SNP density map.

The genomes of these ten *Euonymus* plastomes ranged from 157,263 bp to 157,702 bp in size, with a quadripartite structure that is typical of plastomes in most plant species (Fig 1, Table 1). Each plastome consisted of a large single copy (LSC) region ranging from 85,855 bp to 86,532 bp, a small single copy (SSC) region of 18,315 bp to 18,708 bp, and two inverted repeats (IR) sequences ranging from 26,322 bp to 26,719 bp. Gene content and gene order were similar throughout the analyzed species. All ten *Euonymus* plastomes contained 87–88 protein-coding genes, 37 tRNA genes, and 8 rRNA genes. The gene numbers differed among species due to the *rps19* gene. The *rps*19 gene was located in the LSC region of the *E*. *hamiltonianus*, *E*. *europaeus* and *E*. *szechuanensis* plastomes, whereas the gene was located in the IR region of the *E*. *japonicus*, *E*. *fortunei* and *E*. *schensianus* plastomes. Thus, species with *rps*19 located in the IR region had one more copy of the gene, while others only had one copy in the LSC region. Twenty-two genes were identified as multi-exon genes containing introns (S1 Table).

**Fig 2:**
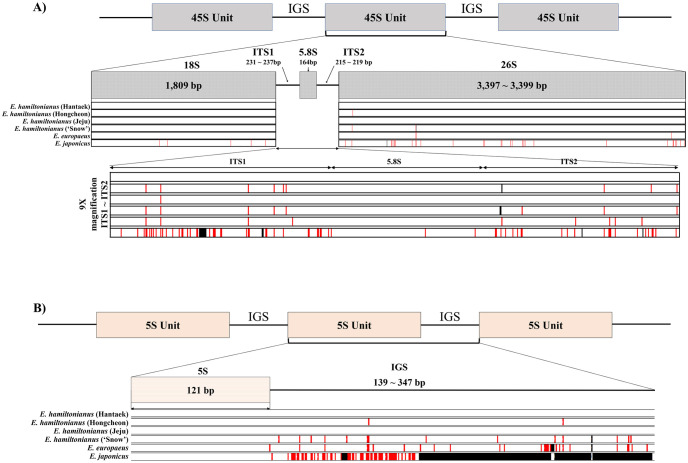
Genomic structures and diversity of rDNAs in *Euonymus*. A and B show the structures of 45S rDNA and 5S rDNA, respectively. Variations are marked based on *E*. *hamiltonianus* (Hantaek) as a reference and are highlighted by red and black lines. Red and black lines indicate SNPs and InDels, respectively.

The six 45S rDNA sequences varied in length from 5,822 bp to 5,825 bp (Fig 2, Table 2). The lengths of specific regions of the six 45S rDNA sequences were similar: 1,809 bp for 18S regions, 231 bp to 237 bp for ITS1 regions, 164 bp for 5.8S regions, 215 bp to 219 bp for ITS2 regions, and 3,397 bp to 3,399 bp for 26S rDNA regions. Even though the sizes of 45S rDNAs were quite similar, *E*. *japonicus* showed a relatively divergent 45S rDNA sequence.

The 5S rDNA transcription unit sequences of *E*. *hamiltonianus* and *E*. *japonicus* were identical, and that of *E*. *europaeus* contained a single nucleotide polymorphism (SNP) (Fig 2). By contrast, the sequences of intergenic spacer (IGS) regions significantly varied. The total length of the 5S unit and the IGS region ranged from 260 bp to 468 bp. Among the six *Euonymus* lines, *E*. *japonicus* had an exceptionally short IGS region compared to the five others, including *E*. *hamiltonianus* and *E*. *europaeus*. Furthermore, the assembled 5S rDNA repeat array existed independently from 45S rDNA, indicating that each nrDNA unit is independently repeated.

### Plastome diversity among *Euonymus* lines

We evaluated the genome structures and sequence similarity of the ten *Euonymus* plastomes. Hundreds of SNPs (71–270) and InDels (70–213) were identified among the four *E*. *hamiltonianus* lines (Table 3). Each *E*. *hamiltonianus* accession showed unique patterns of SNP distribution (Fig 1 and S1 Fig). The IR regions were more conserved than the LSC and SSC regions. The sequences in protein-coding regions showed high similarity compared with those in non-coding regions. In particular, protein-coding genes in the IR region showed relatively high interspecies similarity than those in SC regions.

**Table 3:** Numbers of SNPs and InDels between the plastomes and 45S rDNA sequences of the four *E*. *hamiltonianus* accessions.

InDel	*E*. *hamiltonianus* (Hantaek)	*E*. *hamiltonianus* (Hongcheon)	*E*. *hamiltonianus* (Jeju)	*E*. *hamiltonianus* (’Snow’)
SNP				
*E*. *hamiltonianus* (Hantaek)	-	202/1	206/0	213/2
*E*. *hamiltonianus* (Hongcheon)	247/10^#^	-	70/1	100/2
*E*. *hamiltonianus* (Jeju)	246/1	71/9	-	80/2
*E*. *hamiltonianus* (’Snow’)	270/10	89/6	90/9	-

^**#**^Numbers of variations in plastomes/nrDNA are indicated for each comparison.

**Fig 3:**
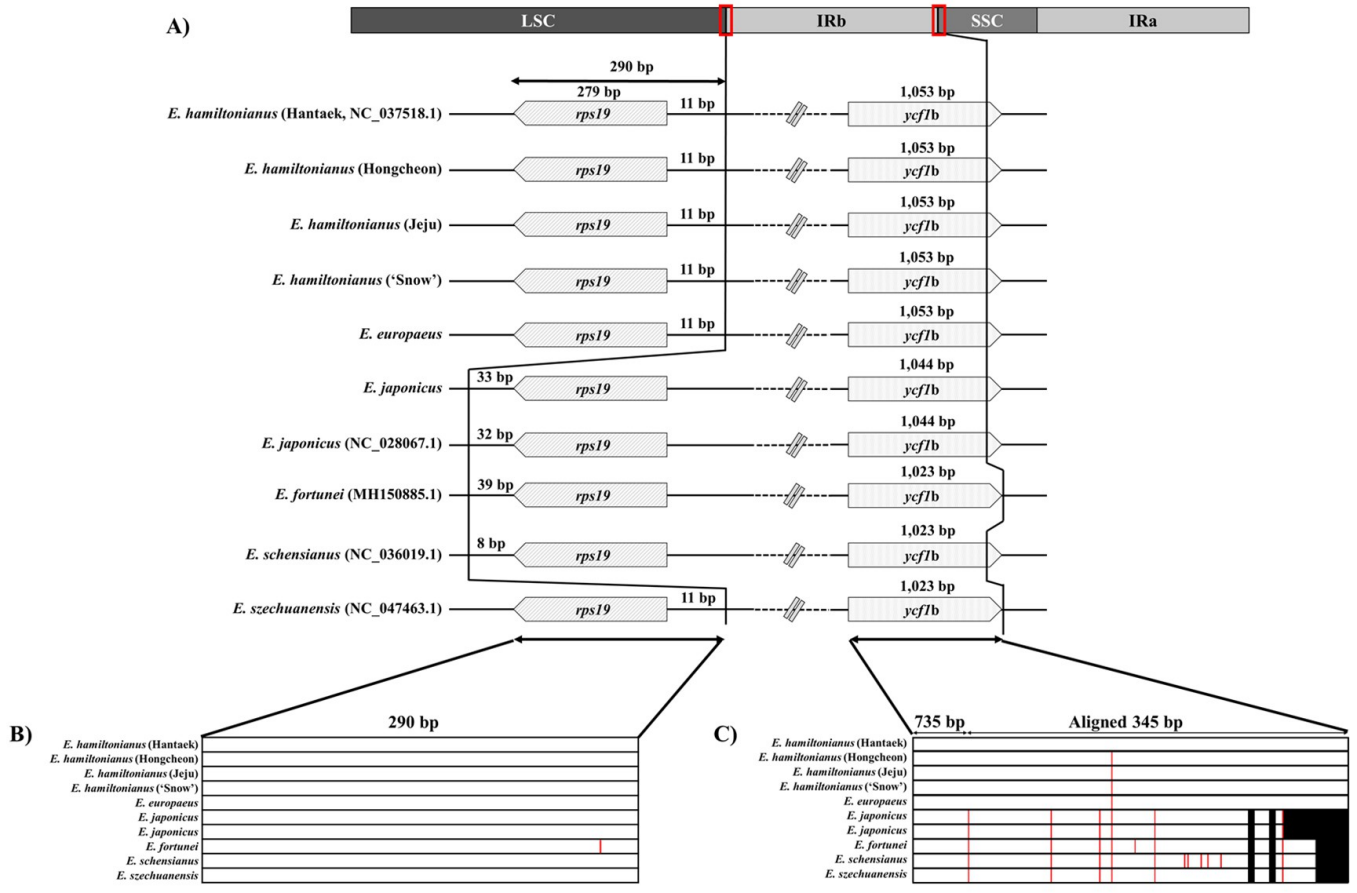
IR junctions in ten *Euonymus* plastomes. A) In every species, *rps19* is 279 bp long and is present at the IR junction. *rps19* is present in the IR in four *Euonymus* individuals and in the LSC in six *Euonymus* individuals. The right IR junctions on the *ycf*1b genes are marked. B) and C) Nucleotide diversity in the left and right junctions, respectively. SNP and InDels are indicated by red and black lines, respectively. All variants are marked based on *E*. *hamiltonianus* (Hantaek) as a reference. The regions upstream of 735 bp in C) have no variants.

Even though structural variations were not detected, slight differences were found at the flanking sequence of the *rps19* gene, which is conserved and duplicated in the IR regions of some *Euonymus* plastomes such as *E*. *japonicus*, *E*. *fortunei*, and *E*. *schensianus* (Fig 3A and 3B). In addition to variation in the *rps19* gene, interspecies variation was found in the junctions of IRb and SSC regions (Fig 3B). There were three types of *ycf1*b genes in this junction based on length: 1,023 bp, 1,044 bp, and 1,053 bp (Fig 3C).

**Fig 4:**
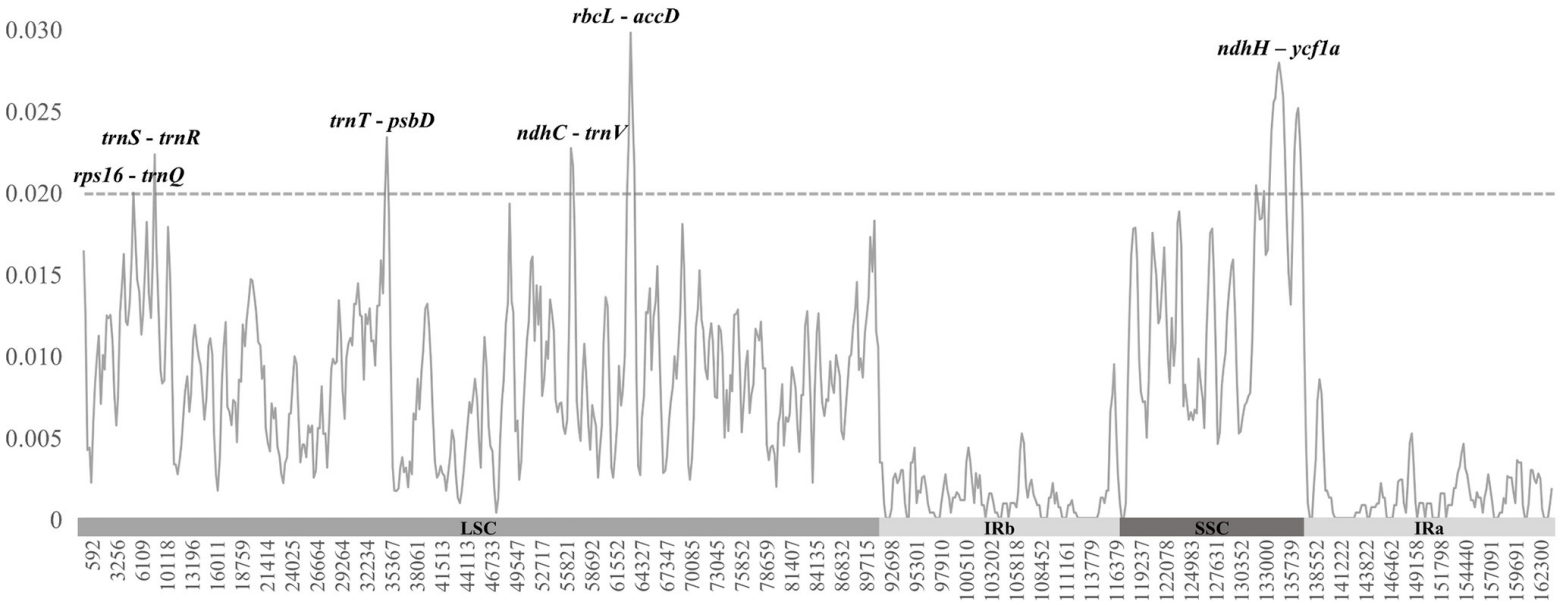
Pi value for each plastome region among the ten *Euonymus* lines. The total pi value among whole plastomes was calculated as 0.00695 using DnaSP with the sliding window method. The sliding window size was 600 bp with a 200 bp sliding length. Six regions were estimated as divergence hotspots among *Euonymus*, as they had higher pi values (> 0.02). IR regions had lower pi values than SC regions (LSC and SSC).

To identify divergence hotspot regions within the *Euonymus* plastome, we detected polymorphic sites and calculated the nucleotide diversity (pi value) (Fig 4). We identified 2,577 polymorphic sites in the ten *Euonymus* plastomes, with an aligned length of 153,495 bp (not including gap regions). Among the polymorphic sites, 1,767 sites (1.15%) were parsimony informative sites, while the remaining 810 sites (0.53%) were singleton sites. The pi value was calculated as 0.00695 throughout the plastome with sliding window methods (window size: 600bp, sliding length: 200bp). The individual windows had pi values ranging from 0 to 0.02993. The highest pi value was observed in the *rbcL*–*accD* region (0.02993). Six intergenic regions (*rps16*–*trnQ*, *trnS*–*trnR*, *trnT–psbD*, *ndhC–trnV*, *rbcL–accD*, and *ndhH*–*ycf1*a) had the higher pi values than the other regions (> 0.02). All six highly diverged regions were located in SC regions: five of these were located in the LSC region, and only one (*ndhH*–*ycf1*a) was located in the SSC region.

### Divergence of protein-coding genes in the *Euonymus* plastid genomes

To investigate sequence divergence of protein-coding genes in the *Euonymus* plastomes, we estimated the ratio of non-synonymous (dN) to synonymous (dS) substitution rates (dN/dS). Based on dN/dS ratios, no gene was estimated to be under positive selection (>1.0), which was similar to most seed plants [52–54]. However, no variation in coding sequence was identified in nine genes, including seven photosynthesis genes (*psbI*, *psaI*, *psbJ*, *psbF*, *psbT*, *psbN*, and *ycf3*) and two ribosomal protein genes (*rpl36* and *rps7*). In addition, 15 genes (*atpH*, *petN*, *psbM*, *psbD*, *psaB*, *psbE*, *petG*, *psaJ*, *rpl33*, *clpP*, *petD*, *infA*, *rps19*, *rpl23*, and *psaC*) contained only synonymous substitution sites. In other words, these 24 genes had conserved protein-coding sequences in the *Euonymus* plastomes. Among the four *E*. *hamiltonianus* plastomes, 51 genes had conserved protein-coding sequences, 33 of which had no nucleotide variations.

### Diversity of 45S and 5S nrDNA in *Euonymus*

Even though the 45S rDNA subunits were similar in length, many variations accumulated among species (Fig 2A). Each ribosome unit (18S, 5.8S, and 26S) had fewer variations than ITS regions. We identified 95 *E*. *japonicus*-specific SNPs and 17 SNPs in *E*. *hamiltonianus* and *E*. *europaeus*. Among the 112 SNPs identified, 5, 3, 34, and 70 were detected in 18S, 5.8S, 26S, and ITS regions, respectively (S2 Table).

The sequences of the 5S rDNA transcription unit were identical in *E*. *hamiltonianus* and *E*. *japonicus*, and one SNP was found in *E*. *europaeus* (Fig 2B). However, the sequences of IGS regions were quite variable. The IGS region was shorter in *E*. *japonicus* (139 bp) than in other species (343 to 347 bp) with abundant SNPs (Fig 2B).

### Phylogenetic analysis of *Euonymus*

**Fig 5:**
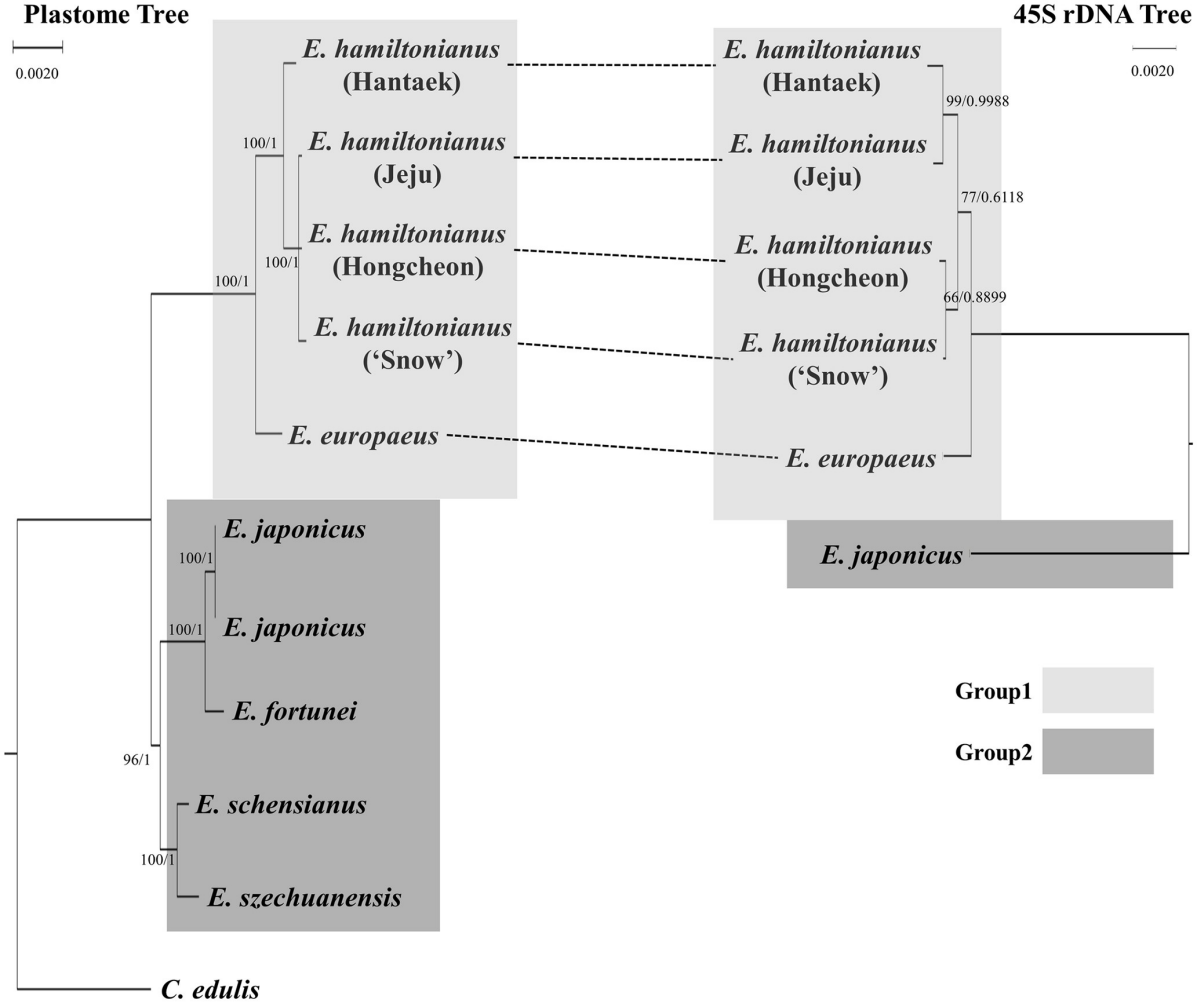
Phylogenetic tree of the ten *Euonymus* accessions based on plastome and 45S rDNA sequences. The tree on the left was drawn using common plastome coding sequences, and the tree on the right was drawn using common 45S rDNA sequences. The phylogenetic trees were constructed using the Bayesian Inference method. Bootstrap (ML method) and posterior probability values greater than 50% were shown. The supporting values separated by slash are bootstrap values and posterior probability, respectively.

The concatenated 77 protein-coding gene sequences were 62,724 bp in aligned length. Based on the supermatrix, the plastome-based phylogenetic tree of *Euonymus* was constructed by using *C*. *edulis* as an outgroup species. The plastome sequences from the ten *Euonymus* plastomes were divided into two groups in the phylogenetic tree (Fig 5 and S2 Fig). Group 1 contained all four accessions of *E*. *hamiltonianus* and *E*. *europaeus*, while Group 2 contained five other *Euonymus* lines (two *E*. *japonicus* accessions, *E*. *fortunei*, *E*. *schensianus*, and *E*. *szechuanensis*). The BI and ML trees showed almost identical topologies (S2 Fig). In Group 1, *E*. *europaeus* was sister to all four *E*. *hamiltonianus* accessions, and an *E*. *hamiltonianus* accession (Hantaek) was sister to the three other *E*. *hamiltonianus* accessions. However, the phylogenetic relationship inferred from nrDNA sequences was inconsistent with the results inferred from plastome sequences (Fig 5). In the nrDNA tree, two *E*. *hamiltonianus* accessions (Hantaek and Jeju) and the two other *E*. *hamiltonianus* accessions (Hongcheon and ‘Snow’) formed independent subclades, with sister relationships (Fig 5), suggesting that cytonuclear discordance could exist in this species. In Group 2, *E*. *japonicus* and *E*. *fortunei* formed a subclade, which was sister to the other subclade of *E*. *schensianus* and *E*. *szechuanensis*. We were not able to reconstruct the phylogenetic relationships of species in Group 2 based on nrDNA data because the nrDNA sequences of *E*. *fortunei*, *E*. *schensianus*, and *E*. *szechuanensis* in Group 2 were not available (Fig 5).

### Marker development for the identification of diverse *Euonymus hamiltonianus* resources

**Fig 6:**
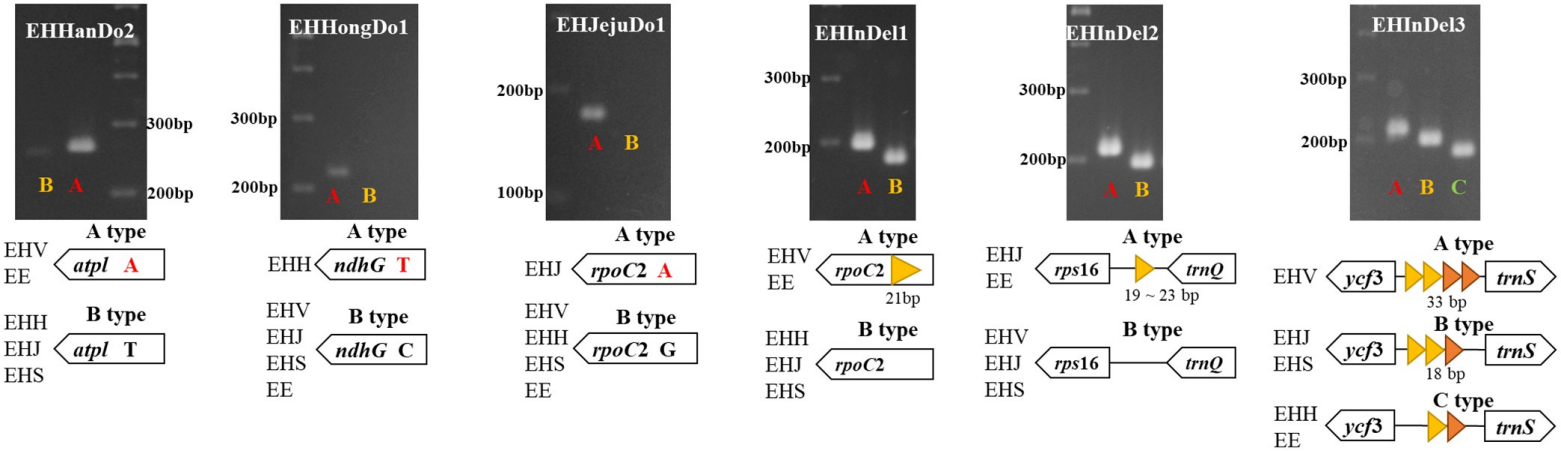
Representative genotypes and scheme used to develop barcoding markers. EHV: *E*. *hamiltonianus* (Hantaek), EHH: *E*. *hamiltonianus* (Hongcheon), EHJ: *E*. *hamiltonianus* (Jeju), EHS: *E*. *hamiltonianus* (‘Snow’), EE: *E*. *europaeus*.

We developed six DNA barcoding markers from three SNPs and three InDel regions in the *E*. *hamiltonianus* plastomes. Three SNP markers were developed from *atpI*, *ndhG*, and *rpoC2* while three InDel markers were developed from *rpoC2*, intergenic regions between *rps*16–*trnQ* and *ycf*3–*trnS* (Fig 6, Table 4).

**Table 4:** Primers used in this study.

Primer	Location	Accession	Product size (bp)	Strand	Primer sequence
EHHanDo2	*atpI*	EHV, EE	270	F	GTTGACCTACTTCCACAGGA
		EHH, EHJ, EHS	-	R	CAATCGCTTTTTATTCCTGAATCG
EHHongDo1	*ndhG*	EHH	232	F	AGGCCCAATCCCAGAAAGTT
		EHV, EHJ, EHS, EE	-	R	GTGTCGAACTCTCCTTTGGG
EHJejuDo1	*rpoC2*	EHJ	184	F	CCGATTTTAGGTGTGGTGGA
		EHV, EHH, EHS, EE	-	R	GGGAGAGTTTCTAAGCCCGA
EHInDel1	*rpoC2*	EHV, EE	209	F	CGAAGTTGCAGGTTTTCCCTT
		EHH, EHJ, EHS	188	R	TTGATACCACCAGGAACGGT
EHInDel2	*rps*16—*trnQ*	EHJ, EE	224	F	AATGGATCGGGAATCGGAGG
		EHV, EHJ, EHS	205	R	TGATCCTAGAAAGATGAGAACATGG
EHInDel3	*ycf*3—*trnS*	EHV	224	F	TGCTCGTGAGAAAACCACCA
		EHJ, EHS	209	R	TCGAAACTACTCCATTTGGTTTGG
		EHH, EE	191		

F: Forward; R: Reverse; EHV: *E*. *hamiltonianus* (Hantaek); EHH: *E*. *hamiltonianus* (Hongcheon); EHJ: *E*. *hamiltonianus* (Jeju); EHS: *E*. *hamiltonianus* (‘Snow’); EE: *E*. *europaeus*.

All six markers were successfully applied to 32 *Euonymus* lines, including 31 *E*. *hamiltonianus* accessions and one *E*. *europaeus* accessions (S4–S9 Figs). Based on the results, the 32 lines were divided into six groups (S3 Fig). Two accessions (28 and 29 in S3 Fig) showed a different genotype from the other accessions in Group 1. Despite the high intraspecies diversity in *E*. *hamiltonianus*, all *E*. *hamiltonianus* accessions were sympatrically separated with their exclusive genotypes (S5 Table). These results suggest that *E*. *hamiltonianus* accessions in Korea adapted to their specific environments and accumulated significant genetic divergence.

## Discussion

### Super-barcoding using complete plastome sequences for species identification

Although phylogenetic studies of the Celastraceae family have been performed using short barcoding regions [5–8], interspecies or intergeneric boundaries in this family have remained unclear due to the limited information provided by the short universal barcoding regions, such as *rbcL* and *matK* genes [24]. Moreover, mitochondrial plastid DNA (MTPT) has been reported in these barcoding genes in many plants [20,55,56]. Therefore, whole plastome sequences were proposed as an alternative source for developing useful markers for super-barcoding [25]. In this study, we obtained the complete plastome and nrDNA sequences of *Euonymus* species for use in species identification. The *Euonymus* species were clearly identified using these sequences, and the newly developed markers obtained in this study showed sufficient performance for the identification of different *E*. *hamiltonianus* accessions. Moreover, we inferred the divergence and evolutionary history of the genus *Euonymus* based on our newly assembled data. Therefore, complete plastomes could provide more precise information for accurate species authentication compared to short barcoding regions.

In this study, we developed six markers to distinguish individuals of *E*. *hamiltonianus* collected from various sources. According to marker validation results, it was possible to divide our *E*. *hamiltonianus* individuals into five groups (S3 Fig). Different marker combinations could be applied to identify the genotype of each group; Hantaek (EHV) genotype: EHHanDo2, EHInDel1, and EHInDel3. Hongcheon (EHH) genotype: EHHongDo1 and EHInDel3. Jeju (EHJ) genotype: EHJejuDo1. Cultivar ‘Snow’ (EHS) genotype:EHInDel2, and EHInDel3. Others: EHJejuDo1, EHInDel2, and EHInDel3. Single markers such as EHHongDo1 and EHInDel3 can be used to identify the Hongcheon genotype and Hantaek genotype, respectively. However, it would be better to use multiple markers to avoid misidentification. We expect that the six markers developed in this study will help to assess genotypes and genetic diversity for conservation of *E*. *hamiltonianus*.

### Nuclear rDNA of *Euonymus*

In this study, we assembled the 45S rDNA and 5S rDNA transcription units with their ITS and IGS regions. The 45S rDNA transcription unit showed higher similarity than the ITS regions in these accessions. In addition, *E*. *japonicus* lines in Group 2 showed highly diverged sequences in their transcription units (Fig 2A). By comparing five newly assembled 45S rDNA sequences, we detected 21 SNPs and 3 InDels. Only seven variants showed homozygosity in all *Euonymus* lines. In the other variants, at least one individual showed heterozygosity (S3 Table). Therefore, caution should be taken when developing authentication markers based on nrDNA sequences. Even when markers are well designed, a heterozygotic position could interfere with the identification and interpretation of the results. A previous study showed that only 10% of non-target sequences could lead to a DNA marker paradox [20].

The complete 5S rDNA sequences showed high similarity among the *Euonymus* species (Fig 2B). However, the IGS sequence of *E*. *japonicus* (Group 2) differed from those of *E*. *hamiltonianus* and *E*. *europaeus* by having a shorter IGS (139 bp) with lower sequence homology. Such variations in the IGS sequence have been reported for other plants [57–60]. For example, differences in 5S rDNA sequences and reductions in repeat length were reported in *Nicotiana tabacum* and *Solanum* species, respectively [58,60].

Considering its sequence characteristics, the 5S rDNA tandem repeat array in *Euonymus* may have undergone a specific evolutionary event. Further research will be needed to elucidate the genomic characteristics and evolutionary history of the nuclear genomes of *Euonymus* species.

### Divergence of the IR junctions in *Euonymus*

The expansion or contraction of IR regions is often observed in land plants [61–64]. Therefore, length variation in IR regions could be considered a common phenomenon in the plastomes of plants. As confirmed in our phylogenetic analysis (Fig 5), the ten *Euonymus* lines could also be divided into two groups. We predicted a divergence event in the *Euonymus* plastomes based on the results of phylogenetic analysis and differences in IR junctions. The *Euonymus* plastomes could be divided into two types based on the location of the *rps19* gene. The plastomes of *E*. *hamiltonianus*, *E*. *europaeus*, and *E*. *szechuanensis* had shorter IR regions, and the *rps19* gene was located in the LSC region (Fig 3), whereas an expansion of the IR containing the *rps19* gene has occurred in the plastomes of *E*. *japonicus*, *E*. *fortunei*, and *E*. *schensianus* (Fig 3). Interestingly, our plastome-based phylogeny indicated that *E*. *szechuanensis* and *E*. *schensianus* formed an independent subclade, with different phylogenetic positions from the four other *Euonymus* species (Fig 5). This result is consistent with an infrageneric classification of the genus *Euonymus* [65]: *E*. *hamiltonianus* and *E*. *europaeus* belong to section *Euonymus*, *E*. *japonicus* and *E*. *fortunei* belong to section *Ilicifolii*, and *E*. *szechuanensis* and *E*. *schensianus* belong to section *Uniloculares*. On the other hand, another junction of IRb and SSC regions showed a similar pattern. The *Euonymus* plastomes were divided into three types based on the lengths of the *ycf1*b gene, which did not correspond to their phylogenetic relationships (Figs 3 and 5). Consequently, it is unclear whether the IR expansion/contraction occurred before or after the split of these three subclades (Fig 3). However, we hypothesize that the IR expansion/contraction event likely occurred recently after the split of these three sections based on the results of phylogenetic analysis and infrageneric classification of the genus *Euonymus*.

### The *ndhE in Euonymus* might be under positive mutational pressure

Relatively higher dS and lower dN have widely been observed in plastid genes from most seed plants, and also in seed-free plants such as lycophytes and ferns [52–54,66,67]. Even though most genes would have undergone purifying selection (dN/dS < 1.0), estimating substitution rates of plastid genes in *Euonymus* is needed to understand the plastome evolution in this genus. As expected, we did not detect any gene under positive selection in our *Euonymus* plastome data. However, two genes (*ndhE* and *rpoC1*) had higher posterior probabilities than the other genes, with a significant p-value (< 0.05) (S4 Table). Both genes were located in the SC regions and detected in *E*. *hamiltonianus*. Most angiosperms contain 11 *ndh* genes which are involved in photosynthesis by producing NADH dehydrogenase subunits [68,69]. Products of these genes (NDH polypeptides) form a thylakoid NDH complex that functions in photosynthetic electron transfer [70]. The loss or pseudogenization of the *ndh* gene family has been reported in many plants [68,71,72]. In the current study, a comparison of the dN values of *ndhE* gene with 75 other genes suggested that *ndhE* might be under positive mutational pressure (S10 Fig). The dN value of the *ndhE* gene was two-times greater than the values of other genes. Therefore, it appears that *ndhE* is under relatively high mutational pressure in the three *E*. *hamiltonianus* accessions. Inspection of more diverse plastomes in *Euonymus* species should help clarify the active role of the *ndhE* gene in the divergence of these species.

## Conclusions

In this study, we documented the complete plastomes of *Euonymus* species and performed comparative analysis. The plastome structures in the *Euonymus* genus were quite similar. However, the divergence of the *Euonymus* plastomes were revealed by comparing IR junctions and calculating pi values. We also confirmed the sequences and structures of nrDNAs in *Euonymus*. Phylogenetic analysis revealed possible cytonuclear discordance between the plastid and nuclear genomes, which we used to infer the times of IR expansion/contraction. The six molecular markers developed in this study will be useful for exploring genetic diversity of *E*. *hamiltonianus* distributed in South Korea. Further studies might help confirm the putative cytonuclear discordance between the organelle and nuclear genomes, as well as the divergence of the *ndhE* gene in *E*. *hamiltonianus* through large-scale data analysis.

## Supporting information

S1 Fig Sequence similarity among *Euonymus* accessions. (DOCX)

S2 Fig Phylogenetic trees based on plastome sequences. (DOCX)

S3 Fig Barcode markers used to identify the 31 *E*. *hamiltonianus* accessions. (DOCX)

S4 Fig Scheme for EHHanDo2 marker development and gel-based analysis. (DOCX)

S5 Fig Scheme for EHHongDo1 marker development and gel-based analysis. (DOCX)

S6 Fig Scheme for EHJejuDo1 marker development and gel-based analysis. (DOCX)

S7 Fig Scheme for EHInDel1 marker development and gel-based analysis. (DOCX)

S8 Fig Scheme for EHInDel2 marker development and gel-based analysis. (DOCX)

S9 Fig Scheme for EHInDel3 marker development and gel-based analysis. (DOCX)

S10 Fig dN trees of *ndhE* and other genes. (DOCX)

S1 Table Plastid gene contents and categories in *Euonymus*. (DOCX)

S2 Table 45S rDNA variants among six *Euonymus* accessions. (DOCX)

S3 Table Variants among the newly assembled Group 1 *Euonymus* collections. (DOCX)

S4 Table Genes showing significant p-values. (DOCX)

S5 Table Marker validation result of 32 *Euonymus* accessions using six markers. (DOCX)

S6 Table Samples information used in this study. (DOCX)

S1 Raw images (PDF)
