# Supplementary material for: Analysis of the complete plastomes and nuclear ribosomal DNAs from Euonymus hamiltonianus and its relatives sheds light on their diversity and evolution
Source: PLoS One. 2022 Oct 5;17(10):e0275590. doi: 10.1371/journal.pone.0275590 (PMC9534445; doi:10.1371/journal.pone.0275590)
Supplement: S1 Fig — (DOCX) [file pone.0275590.s001.docx]

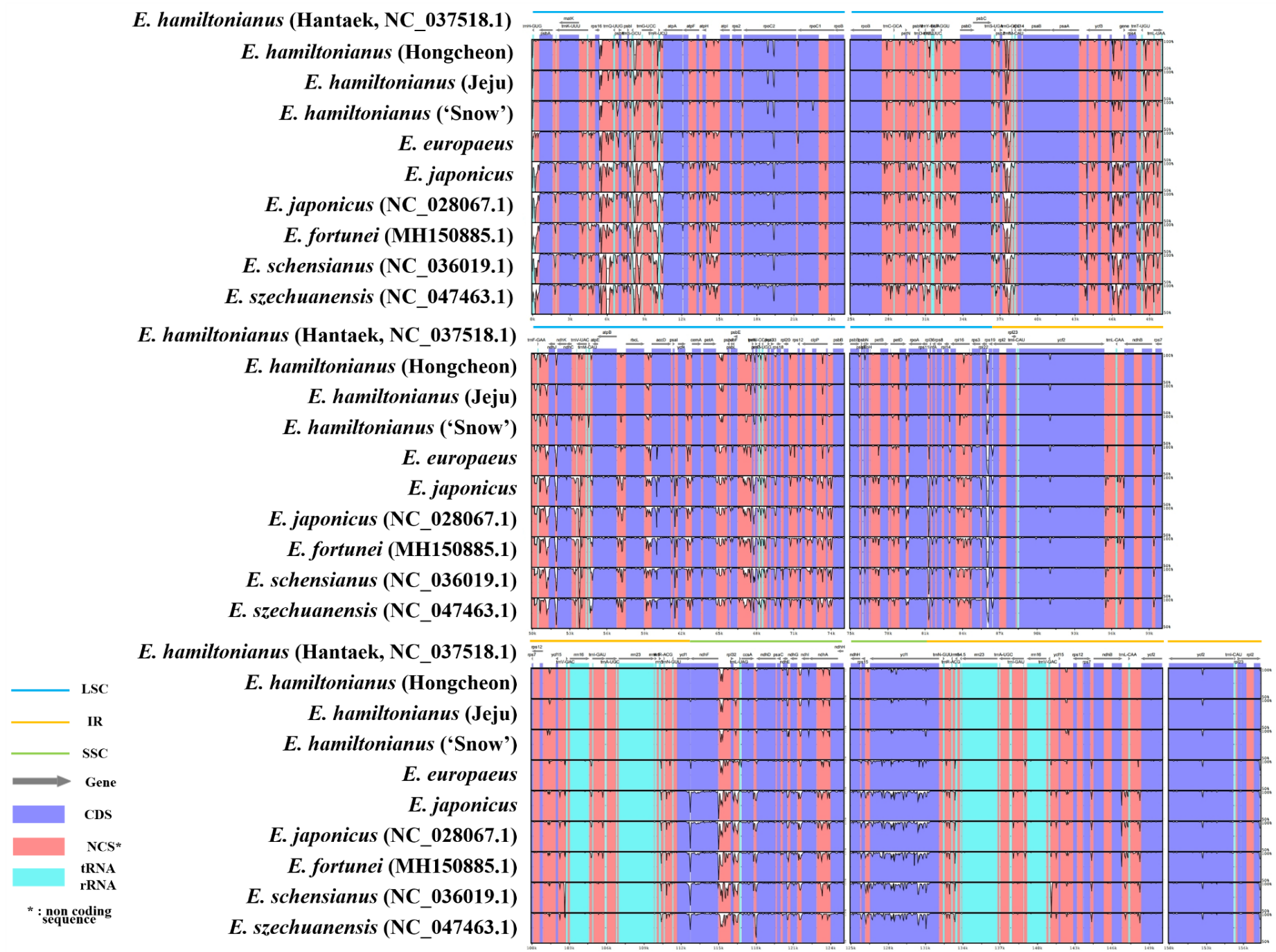


S1 Figure. Sequence similarity among *Euonymus* accessions. mVISTA with the LAGAN alignment method with default parameters (probability threshold 0.5) were used for analysis. *E. hamiltonianus* (Hantaek) was used as a reference.
