# Supplementary material for: Analysis of the complete plastomes and nuclear ribosomal DNAs from Euonymus hamiltonianus and its relatives sheds light on their diversity and evolution
Source: PLoS One. 2022 Oct 5;17(10):e0275590. doi: 10.1371/journal.pone.0275590 (PMC9534445; doi:10.1371/journal.pone.0275590)
Supplement: S2 Fig — (DOCX) [file pone.0275590.s002.docx]

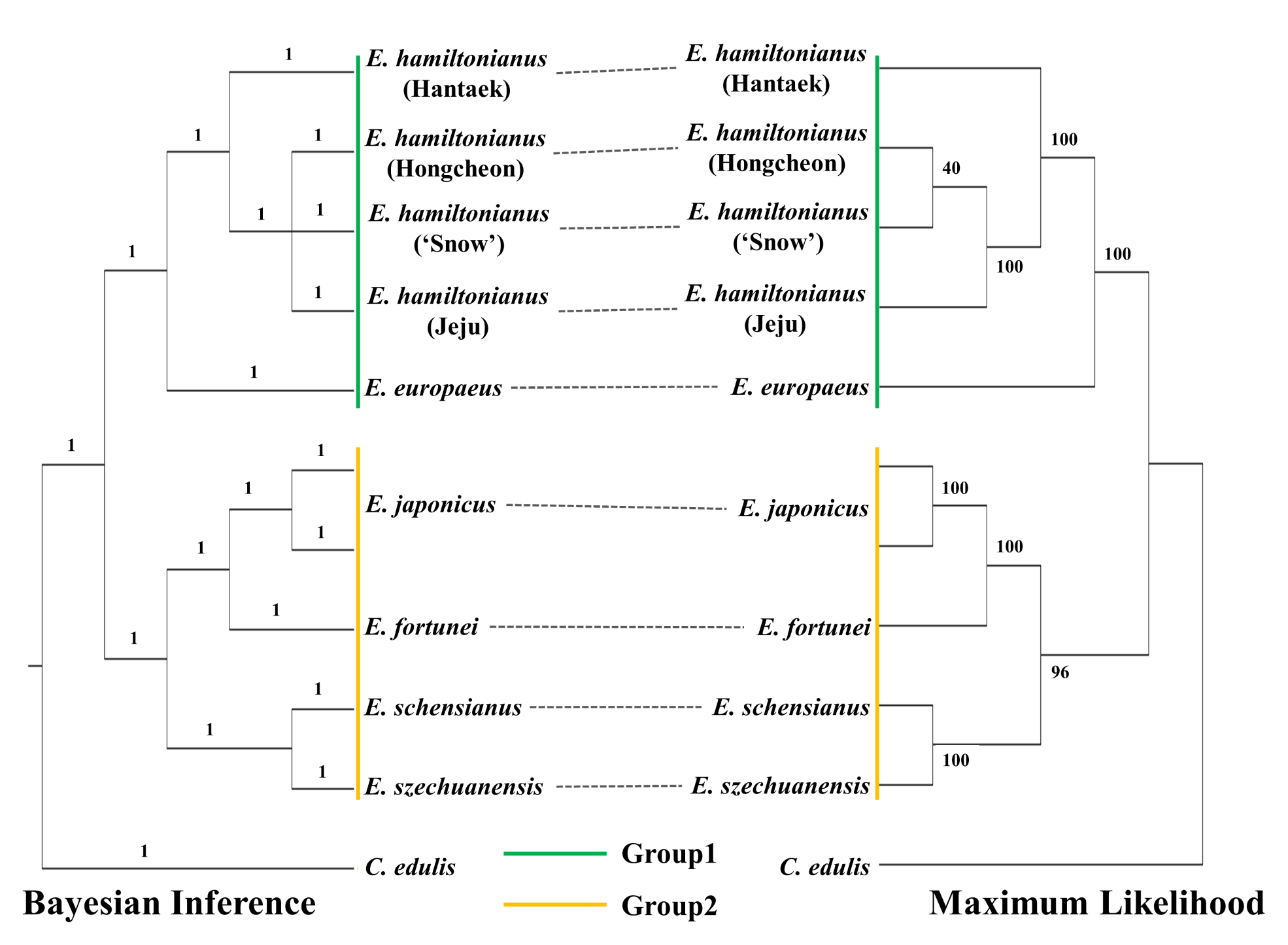


S2 Figure. Phylogenetic trees based on plastome sequences. The tree on the left was drawn using the Bayesian inference method, while the tree on the right was drawn using the Maximum likelihood method. Values on the left tree branches are posterior probabilities. Values on the right tree branches are bootstrap values.
