# Supplementary material for: Analysis of the complete plastomes and nuclear ribosomal DNAs from Euonymus hamiltonianus and its relatives sheds light on their diversity and evolution
Source: PLoS One. 2022 Oct 5;17(10):e0275590. doi: 10.1371/journal.pone.0275590 (PMC9534445; doi:10.1371/journal.pone.0275590)
Supplement: S3 Fig — (DOCX) [file pone.0275590.s003.docx]

**
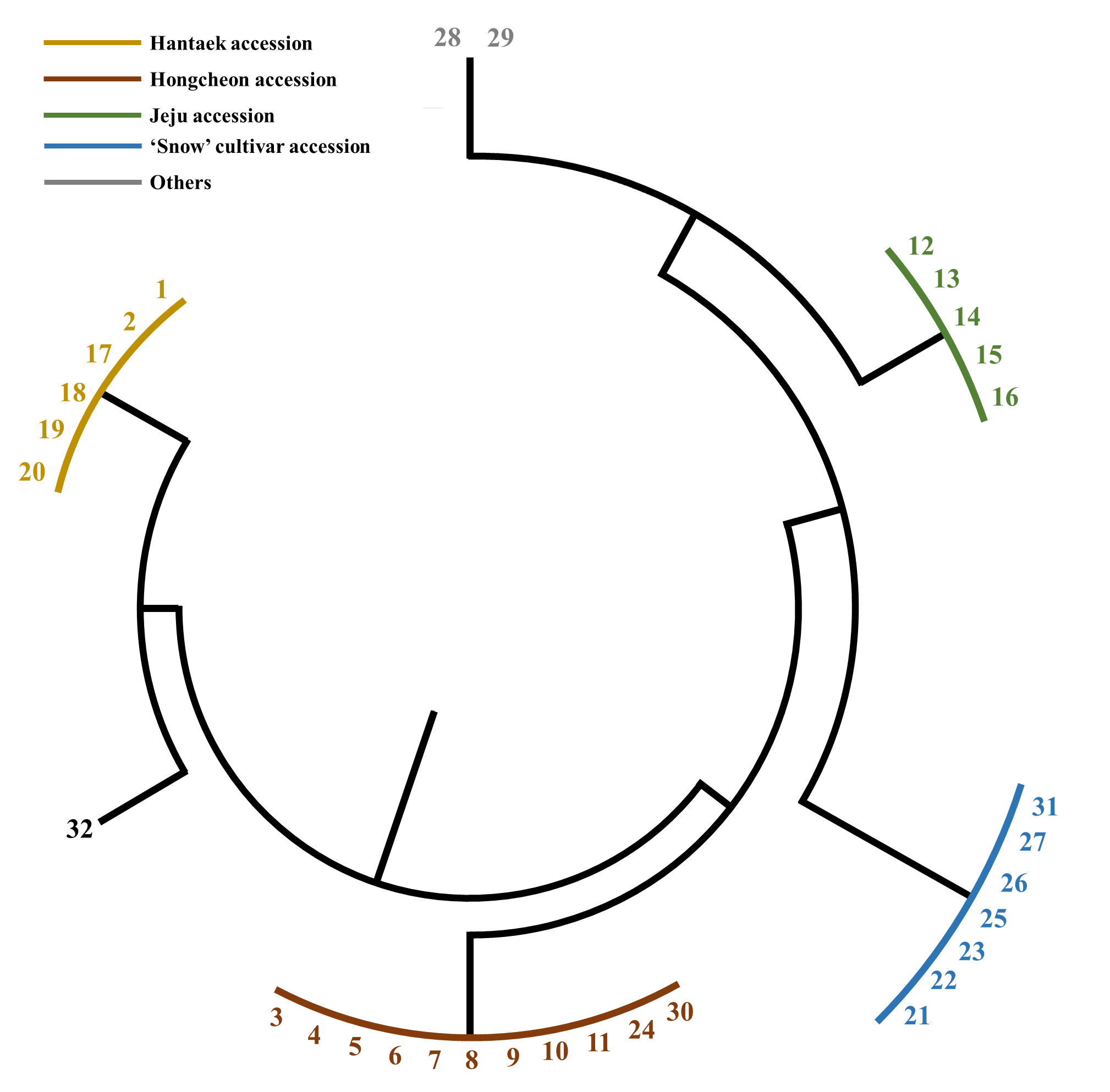
**

S3 Figure. Barcode markers used to identify the 31 *E. hamiltonianus* accessions. 32: *E. europaeus*. All six markers were successfully applied to these accessions.
