# Supplementary figures and images for: Analysis of the complete plastomes and nuclear ribosomal DNAs from Euonymus hamiltonianus and its relatives sheds light on their diversity and evolution
Source: PLoS One. 2022 Oct 5;17(10):e0275590. doi: 10.1371/journal.pone.0275590 (PMC9534445; doi:10.1371/journal.pone.0275590)

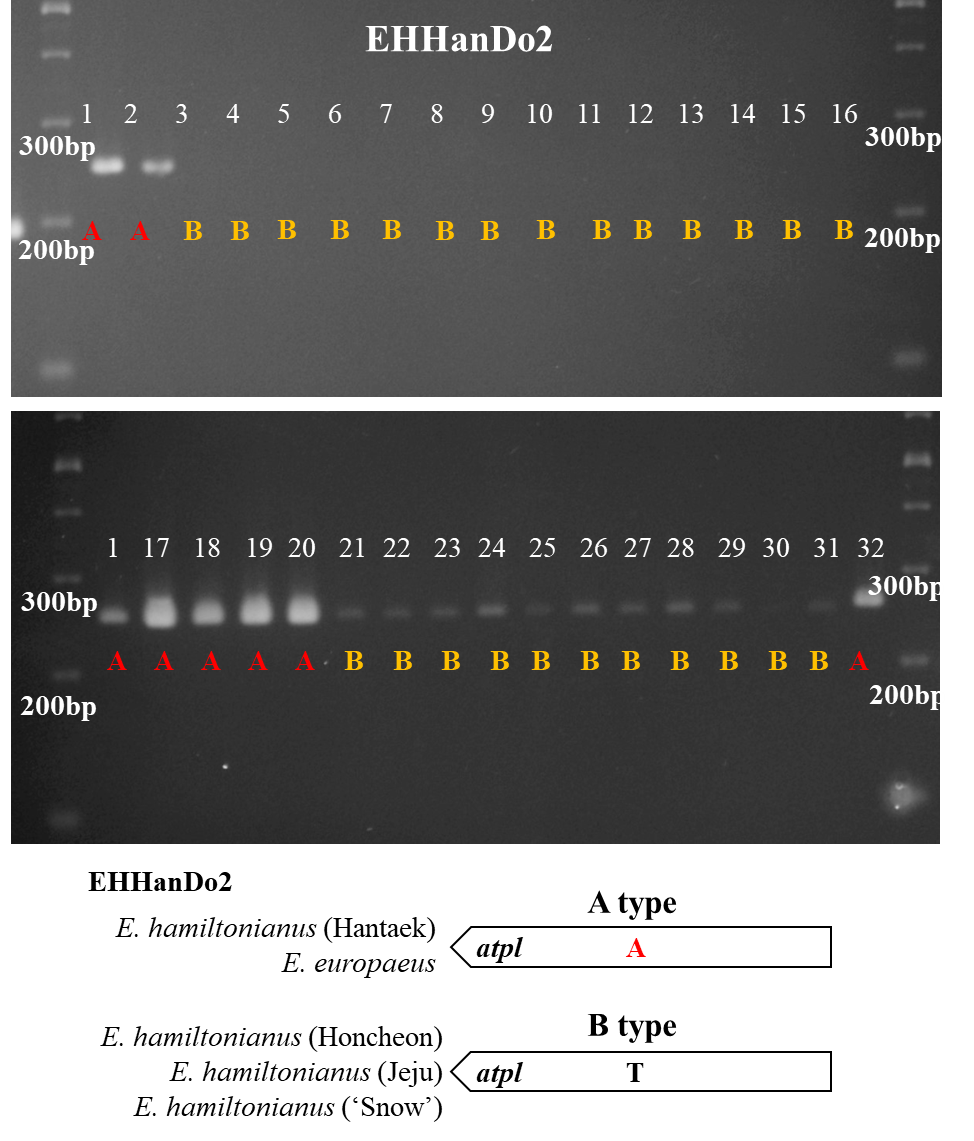


S4 Figure. Scheme for EHHanDo2 marker development and gel-based analysis.

Supplement: S4 Fig — (DOCX) [file pone.0275590.s004.docx]

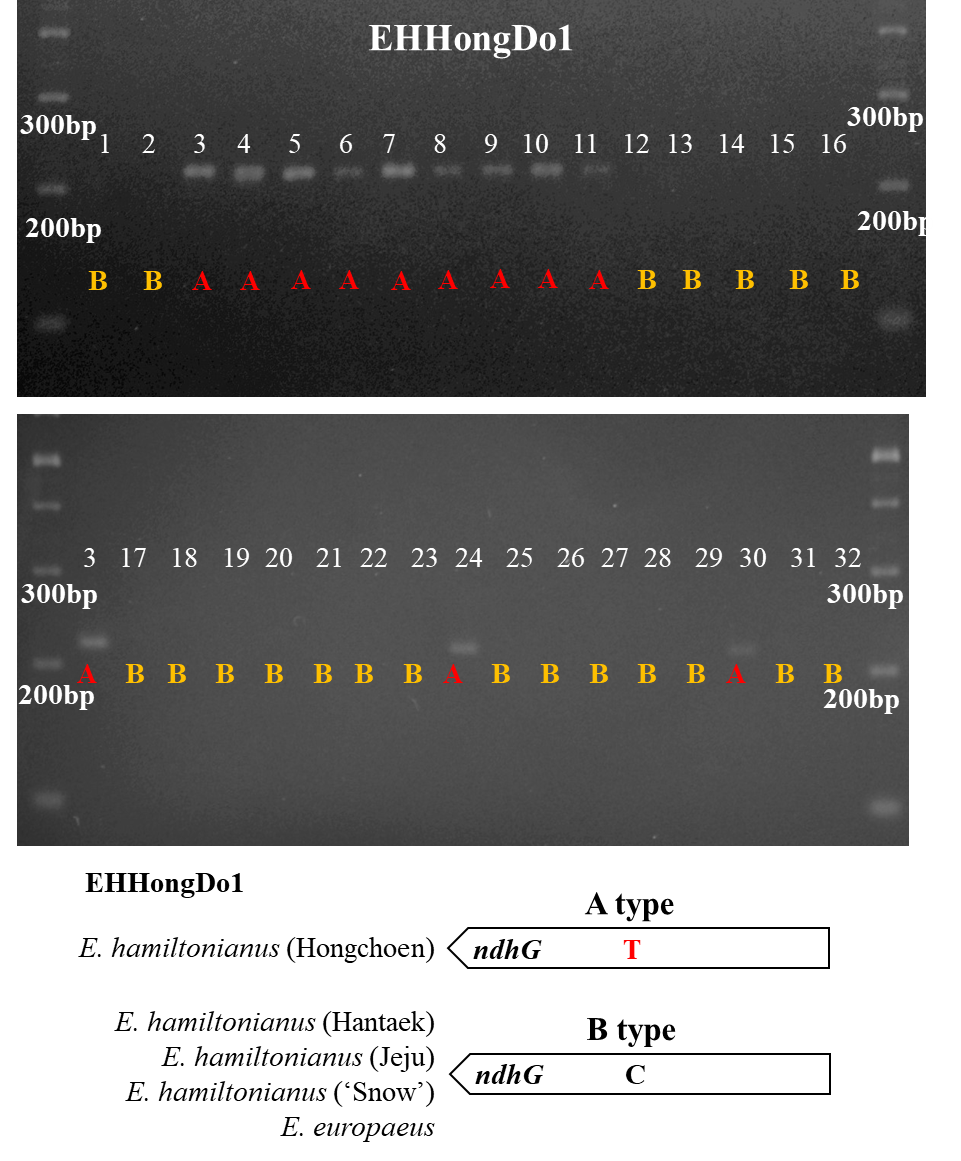


S5 Figure. Scheme for EHHongDo1 marker development and gel-based analysis.

Supplement: S5 Fig — (DOCX) [file pone.0275590.s005.docx]

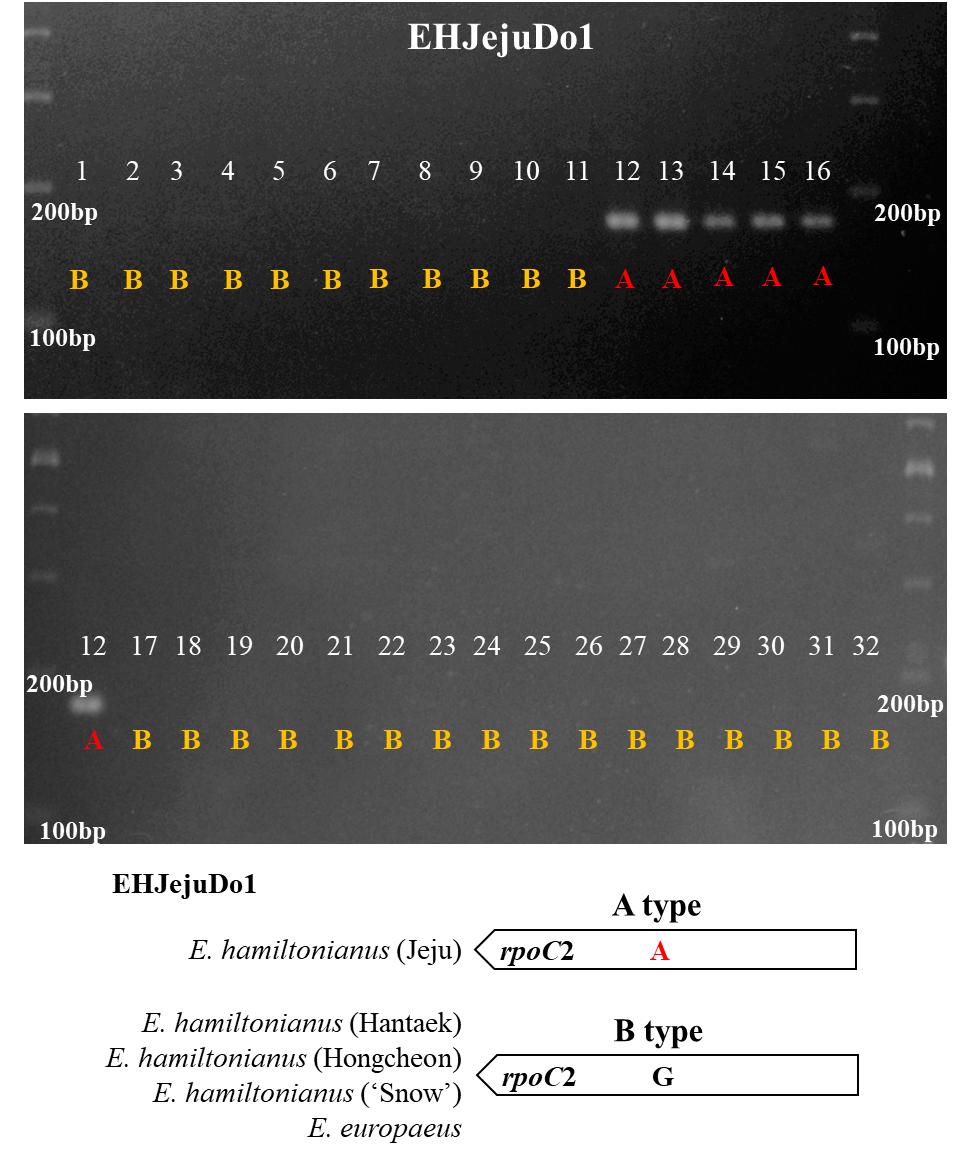


S6 Figure. Scheme for EHJejuDo1 marker development and gel-based analysis.

Supplement: S6 Fig — (DOCX) [file pone.0275590.s006.docx]

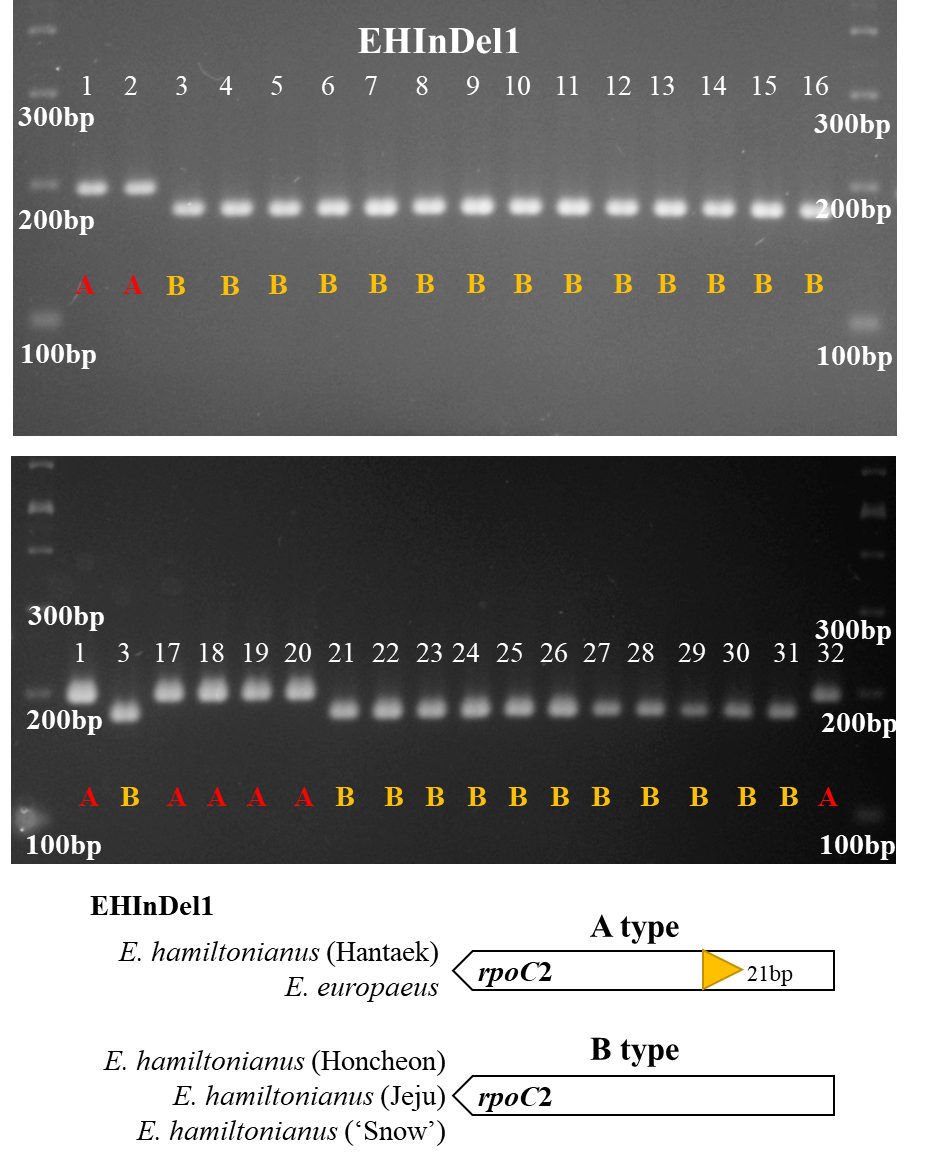


S7 Figure. Scheme for EHInDel1 marker development and gel-based analysis.

Supplement: S7 Fig — (DOCX) [file pone.0275590.s007.docx]

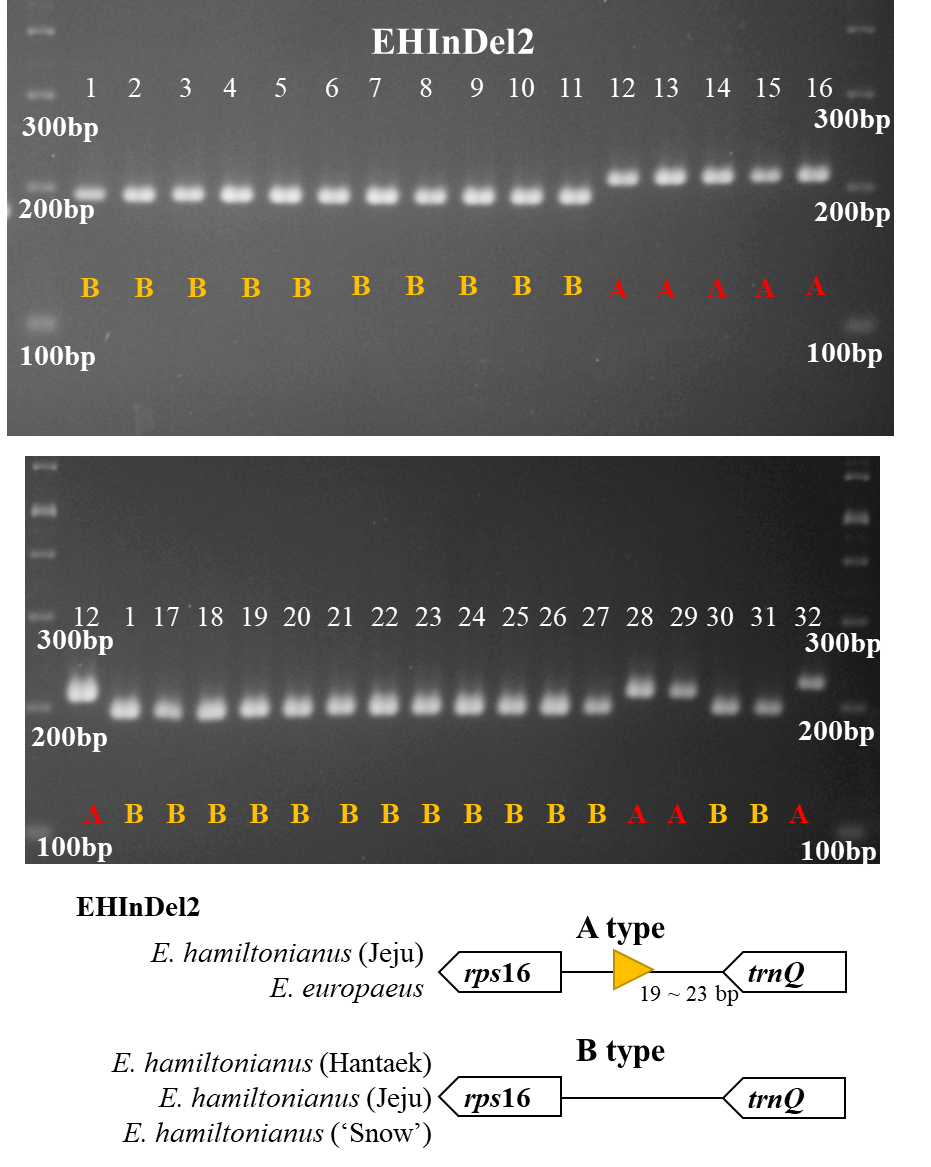


S8 Figure. Scheme for EHInDel2 marker development and gel-based analysis.

Supplement: S8 Fig — (DOCX) [file pone.0275590.s008.docx]

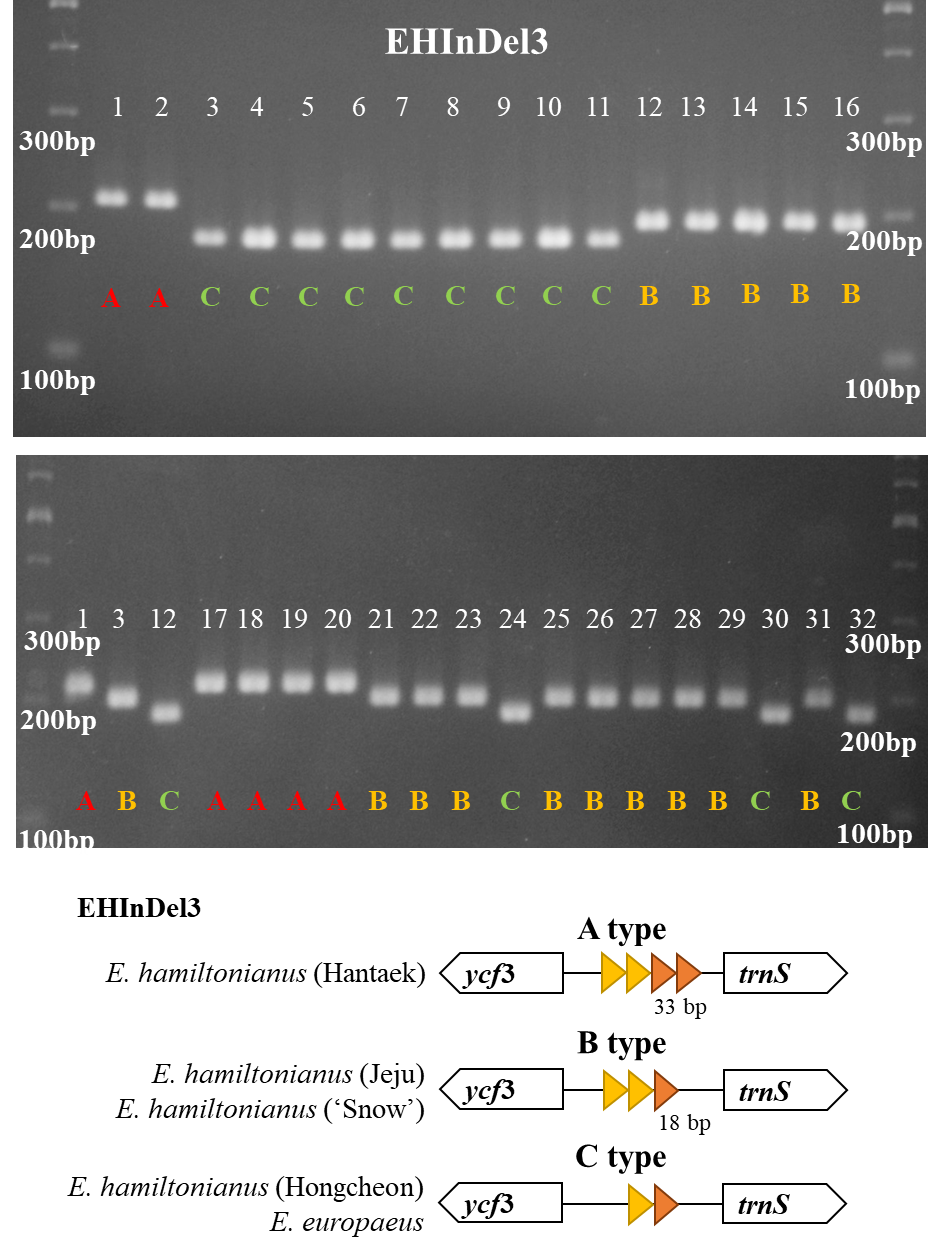


S9 Figure. Scheme for EHInDel3 marker development and gel-based analysis.

Supplement: S9 Fig — (DOCX) [file pone.0275590.s009.docx]
