# Supplementary material for: Analysis of the complete plastomes and nuclear ribosomal DNAs from Euonymus hamiltonianus and its relatives sheds light on their diversity and evolution
Source: PLoS One. 2022 Oct 5;17(10):e0275590. doi: 10.1371/journal.pone.0275590 (PMC9534445; doi:10.1371/journal.pone.0275590)
Supplement: S10 Fig — (DOCX) [file pone.0275590.s010.docx]

**
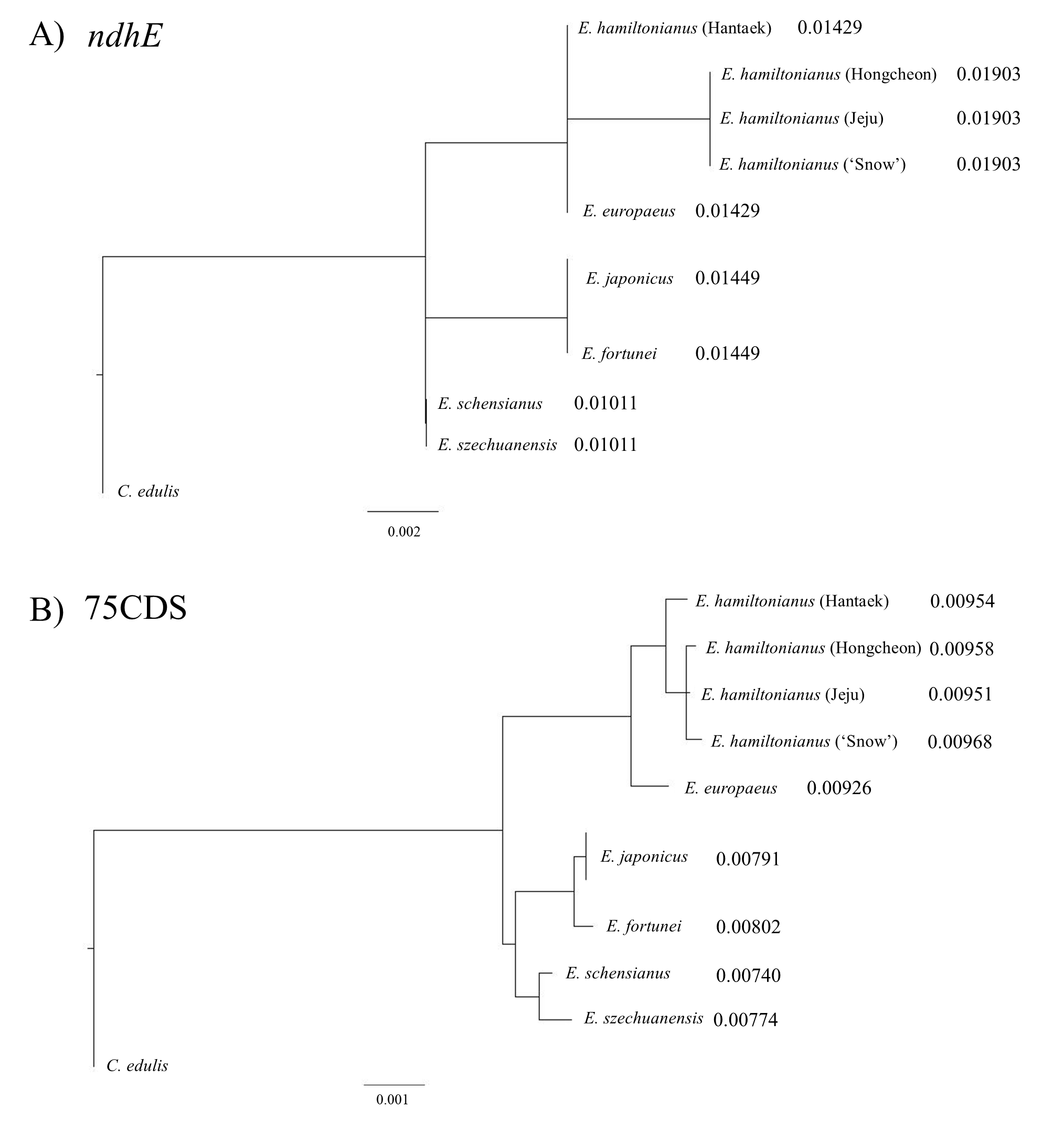
**

S10 Figure. dN trees of *ndhE* and other genes. A) dN tree of *ndhE* gene in each species with dN values. B) dN tree based on the coding sequences of 75 other genes with dN values for each species.
