# Supplementary material for: Analysis of the complete plastomes and nuclear ribosomal DNAs from Euonymus hamiltonianus and its relatives sheds light on their diversity and evolution
Source: PLoS One. 2022 Oct 5;17(10):e0275590. doi: 10.1371/journal.pone.0275590 (PMC9534445; doi:10.1371/journal.pone.0275590)
Supplement: S1 Table — (DOCX) [file pone.0275590.s011.docx]

S1 Table. Plastid gene contents and categories in *Euonymus*.

| Gene Category | Gene Group | Genes |
| --- | --- | --- |
| Photosynthetic apparatus | Photosystem I | *psaA, psaB, psaC, psaI, psaJ* |
|  | Photosystem II | *psbA, psbB, psbC, psbD, psbE, psbF, psbH, psbI, psbJ, psbK, psbL, psbM, psbN, psbT, psbZ* |
|  | Cytochrome b/f complex | *petA, petB*^1)^*, petD*^1)^*, petG, petL, petN* |
|  | ATP synthase | *atpA, atpB, atpE, atpF*^1)^*, atpH, atpI* |
|  | NADH dehydrogenase | *ndhA*^1)^*, ndhB*^1),3)^*, ndhC, ndhD, ndhE, ndhF, ndhG, ndhH, ndhI, ndhJ, ndhK* |
|  | RubisCO large subunit | *rbcL* |
| RNA genes | ribosomal proteins (LSU) | *rpl14, rpl16*^1)^*, rpl2*^1),3)^*, rpl20, rpl22, rpl23*^3)^*, rpl32, rpl33, rpl36* |
|  | ribosomal proteins (SSU) | *rps11, rps12*^2),3),5)^*, rps14, rps15, rps16, rps18, rps19*^4)^*, rps2, rps3, rps4, rps7*^3)^*, rps8* |
|  | RNA polymerase | *rpoA, rpoB, rpoC1*^1)^*, rpoC2* |
|  | ribosomal RNAs | *rrn16*^3)^*, rrn23*^3)^*, rrn4.5*^3)^*, rrn5*^3)^ |
|  | transfer RNAs | *trnA-UGC*^1),3)^*, trnC-GCA, trnD-GUC, trnE-UUC, trnF-GAA, trnfM-CAU, trnG-GCC, trnG-UCC*^1)^*, trnH-GUG, trnI-CAU*^3)^*, trnI-GAU*^1),3)^*, trnK-UUU*^1)^*, trnL-CAA*^3)^*, trnL-UAA*^1)^*, trnL-UAG, trnM-CAU, trnN-GUU*^3)^*, trnP-UGG, trnQ-UUG, trnR-ACG*^3)^*, trnR-UCU, trnS-GCU, trnS-GGA, trnS-UGA, trnT-GGU, trnT-UGU, trnV-GAC*^3)^*, trnV-UAC*^1)^*, trnW-CCA, trnY-GUA* |
| Others | Acetyl-CoA carboxylase | *accD* |
|  | c-type cytochrome synthesis | *ccsA* |
|  | Envelop membrane protein | *cemA* |
|  | ATP-dependent clp protease | *clpP*^2)^ |
|  | Translation initiation factor | *infA* |
|  | maturase | *matK* |
|  | hypothetical chloroplast reading frames (*ycf*) | *ycf1*^3)^*, ycf2*^3)^*, ycf3*^2)^*, ycf4, ycf15*^3)^ |

1) One intron, 2) two introns, 3) two copies in the IR, 4) two copies in *E. japonicus, E. fortunei*, and *E. schensianus*, 5) trans-splicing
