# Supplementary material for: Analysis of the complete plastomes and nuclear ribosomal DNAs from Euonymus hamiltonianus and its relatives sheds light on their diversity and evolution
Source: PLoS One. 2022 Oct 5;17(10):e0275590. doi: 10.1371/journal.pone.0275590 (PMC9534445; doi:10.1371/journal.pone.0275590)
Supplement: S2 Table — (DOCX) [file pone.0275590.s012.docx]

S2 Table. 45S rDNA variants among six *Euonymus* accessions.

| **InDel**  **SNP** | | ***E. hamiltonianus* (Hantaek)** | | | ***E. hamiltonianus* (Hongcheon)** | | ***E. hamiltonianus* (Jeju)** | | | ***E. hamiltonianus* (‘Snow’)** | | | ***E. europaeus*** | | | ***E. japonicus*** | |
| --- | --- | --- | --- | --- | --- | --- | --- | --- | --- | --- | --- | --- | --- | --- | --- | --- | --- |
| ***E. hamiltonianus* (Hantaek)** | | - | | | 1 | | 0 | | | 2 | | | 0 | | | 6 | |
| ***E. hamiltonianus* (Hongcheon)** | | 10 | | | - | | 1 | | | 2 | | | 1 | | | 6 | |
| ***E. hamiltonianus* (Jeju)** | | 1 | | | 9 | | - | | | 2 | | | 0 | | | 6 | |
| ***E. hamiltonianus* (‘Snow’)** | | 10 | | | 6 | | 9 | | | - | | | 2 | | | 7 | |
| ***E. europaeus*** | | 12 | | | 15 | | 11 | | | 13 | | | - | | | 5 | |
| ***E. japonicus*** | | 101 | | | 100 | | 100 | | | 99 | | | 102 | | | - | |
|  | | |  | | |  | | |  | |  | | |  | | |  |
| **Regions**  **Variations Type** | **18S** | | | **ITS1** | | | | **5.8S** | | | | **ITS2** | | | **26S** | | |
| **SNP/InDel** | 5/0 | | | 42/2 | | | | 3/0 | | | | 28/4 | | | 34/2 | | |

There were a total of 112 SNPs and 9 InDels.
