# Supplementary material for: Analysis of the complete plastomes and nuclear ribosomal DNAs from Euonymus hamiltonianus and its relatives sheds light on their diversity and evolution
Source: PLoS One. 2022 Oct 5;17(10):e0275590. doi: 10.1371/journal.pone.0275590 (PMC9534445; doi:10.1371/journal.pone.0275590)
Supplement: S3 Table — (DOCX) [file pone.0275590.s013.docx]

S3 Table. Variants among the newly assembled Group 1 *Euonymus* collections.

| Location | Aligned Position | Variant Type | *E. hamiltonianus* (Hantaek) | *E. hamiltonianus* (Hongcheon) | *E. hamiltonianus* (Jeju) | *E. hamiltonianus* ('Snow') | *E. europaeus* |
| --- | --- | --- | --- | --- | --- | --- | --- |
| ITS1 | 1848 | SNP | **T/A** | **A/T** | **T/A** | A/A | A/A |
|  | 1864 | SNP | **A/C** | **C/A** | **C/A** | C/C | C/C |
|  | 1959 | SNP | **T/C** | **C/T** | **T/C** | C/C | C/C |
|  | 1987 | SNP | **T/C** | **C/T** | **T/C** | C/C | T/T |
|  | 1997 | SNP | **T/C** | **C/T** | **T/C** | T/T | T/T |
|  | 2000 | SNP | **T/C** | **C/T** | **T/C** | C/C | T/T |
|  | 2007 | SNP | G/G | G/G | G/G | G/G | A/A |
| ITS2 | 2229 | InDel | **-/C** | -/- | -/- | **C/-** | -/- |
|  | 2230 | InDel | -/- | -/- | -/- | **C/-** | -/- |
|  | 2231 | InDel | C/C | -/- | **C/-** | C/C | C/C |
|  | 2234 | SNP | C/C | C/C | C/C | C/C | T/T |
|  | 2256 | SNP | T/T | **T/C** | **T/C** | C/C | T/T |
|  | 2314 | SNP | C/C | C/C | C/C | C/C | T/T |
|  | 2345 | SNP | **G/A** | **A/G** | **G/A** | A/A | G/G |
|  | 2351 | SNP | G/G | G/G | G/G | G/G | T/T |
|  | 2357 | SNP | A/A | A/A | A/A | A/A | **G/A** |
|  | 2386 | SNP | G/G | G/G | G/G | G/G | **C/G** |
|  | 2396 | SNP | **C/A** | **A/C** | **C/A** | C/C | C/C |
|  | 2424 | SNP | **A/G** | **G/A** | **A/G** | G/G | A/A |
| 26S | 2558 | SNP | **C/A** | **C/A** | **C/A** | A/A | C/C |
|  | 2564 | SNP | **C/T** | **T/C** | C/C | C/C | C/C |
|  | 3173 | SNP | C/C | C/C | C/C | G/G | G/G |
|  | 3178 | SNP/InDel | G/G | G/G | G/G | -/- | C/C |
|  | 5635 | SNP | G/G | G/G | G/G | G/G | A/A |

Heterozygous aligned positions are marked by red, bold letter. Heterozygotic positions were confirmed by raw reads mapping to the assembled sequences. Mapping conditions were length similarity 90% and sequence similarity 90%. Heterozygotic positions sharing > 5% of the position depth are presented in this table.
