# Supplementary material for: Analysis of the complete plastomes and nuclear ribosomal DNAs from Euonymus hamiltonianus and its relatives sheds light on their diversity and evolution
Source: PLoS One. 2022 Oct 5;17(10):e0275590. doi: 10.1371/journal.pone.0275590 (PMC9534445; doi:10.1371/journal.pone.0275590)
Supplement: S4 Table — (DOCX) [file pone.0275590.s014.docx]

S4 Table. Genes showing significant p-values.

| Species | Gene | BEB | P-value |
| --- | --- | --- | --- |
| *E. hamiltonianus*  (Hongcheon, Jeju, and 'Snow') | *ndhE* | 0.799 | 0.0125476 |
| *E. hamiltonianus*  (Hantaek, Hongcheon, Jeju, and ‘Snow’) | *rpoC*1 | 0.795 | 0.0278715 |
| *Euonymus* | *rps19* | 0.516 | 0.0157689 |
| *E. japonicus* and *E. fortunei* | *ccsA* | - | 0.0421213 |

BEB: Bayes Empirical Bayes
