# Supplementary material for: Analysis of the complete plastomes and nuclear ribosomal DNAs from Euonymus hamiltonianus and its relatives sheds light on their diversity and evolution
Source: PLoS One. 2022 Oct 5;17(10):e0275590. doi: 10.1371/journal.pone.0275590 (PMC9534445; doi:10.1371/journal.pone.0275590)
Supplement: S5 Table — (DOCX) [file pone.0275590.s015.docx]

S5 Table. Marker validation result of 32 *Euonymus* accessions using six markers.

| No. | Collected From | Marker Validation Results | | | | | |
| --- | --- | --- | --- | --- | --- | --- | --- |
|  |  | EHHanDo2 | EHHongDo1 | EHJejuDo1 | EHInDel1 | EHInDel2 | EHInDel3 |
| 1 | HTB | A | B | B | A | B | A |
| 2 | HTB | A | B | B | A | B | A |
| 3 | Hongcheon | B | A | B | B | B | C |
| 4 | Hongcheon | B | A | B | B | B | C |
| 5 | Hongcheon | B | A | B | B | B | C |
| 6 | Hongcheon | B | A | B | B | B | C |
| 7 | Hongcheon | B | A | B | B | B | C |
| 8 | Hongcheon | B | A | B | B | B | C |
| 9 | Hongcheon | B | A | B | B | B | C |
| 10 | Hongcheon | B | A | B | B | B | C |
| 11 | Hongcheon | B | A | B | B | B | C |
| 12 | Jeju | B | B | A | B | A | B |
| 13 | Jeju | B | B | A | B | A | B |
| 14 | Jeju | B | B | A | B | A | B |
| 15 | Jeju | B | B | A | B | A | B |
| 16 | Jeju | B | B | A | B | A | B |
| 17 | HTB | A | B | B | A | B | A |
| 18 | HTB | A | B | B | A | B | A |
| 19 | HTB | A | B | B | A | B | A |
| 20 | HTB | A | B | B | A | B | A |
| 21 | Jeocheon | B | B | B | B | B | B |
| 22 | Jeocheon | B | B | B | B | B | B |
| 23 | Boeun | B | B | B | B | B | B |
| 24 | Hoenseong | B | A | B | B | B | C |
| 25 | HTB | B | B | B | B | B | B |
| 26 | HTB | B | B | B | B | B | B |
| 27 | HTB | B | B | B | B | B | B |
| 28 | HTB | B | B | B | B | A | B |
| 29 | HTB | B | B | B | B | A | B |
| 30 | HTB | B | A | B | B | B | C |
| 31 | HTB | B | B | B | B | B | B |
| 32 | HTB | A | B | B | A | A | C |

HTB: Hantaek Botanical Garden, Yongin, Gyeonggi-do; Hongcheon: Hongcheon district, Gangwon-do; Jeju: Aewol-eup, Jeju-si, Jeju-do; Jeochoen: Jecheon-si, Chungcheongbuk-do; Boeun: Boeun-gun, Chungcheongbuk-do; Hoenseong: Hoengseong-gun, Gangwon-do
