# Supplementary material for: Analysis of the complete plastomes and nuclear ribosomal DNAs from Euonymus hamiltonianus and its relatives sheds light on their diversity and evolution
Source: PLoS One. 2022 Oct 5;17(10):e0275590. doi: 10.1371/journal.pone.0275590 (PMC9534445; doi:10.1371/journal.pone.0275590)
Supplement: S6 Table — (DOCX) [file pone.0275590.s016.docx]

S6 Table. Samples information used in this study.

| No. | Species | Voucher^d^ | Collected From | Collected and identified by |
| --- | --- | --- | --- | --- |
| 1 | *E. hamiltonianus*^a^ | IM160919-2 | Hantaek Botanical Gaden, Baegam-myeon, Cheoin-gu, Yongin-si, Gyeonggi province | Jung wha Kang |
| 2 | *E. hamiltonianus* | IM190628-1 | Hantaek Botanical Gaden, Baegam-myeon, Cheoin-gu, Yongin-si, Gyeonggi province | Jung wha Kang |
| 3 | *E. hamiltonianus*^a^ | IM190717-1 | Nae-myeon, Hongcheon-gun, Gangwon province | Hyun Ok Yang |
| 4 | *E. hamiltonianus* | IM190603-2 | Nae-myeon, Hongcheon-gun, Gangwon province | Hyun Ok Yang |
| 5 | *E. hamiltonianus* | IM190603-3 | Nae-myeon, Hongcheon-gun, Gangwon province | Hyun Ok Yang |
| 6 | *E. hamiltonianus* | IM190603-4 | Nae-myeon, Hongcheon-gun, Gangwon province | Hyun Ok Yang |
| 7 | *E. hamiltonianus* | IM190603-5 | Nae-myeon, Hongcheon-gun, Gangwon province | Hyun Ok Yang |
| 8 | *E. hamiltonianus* | IM190603-6 | Nae-myeon, Hongcheon-gun, Gangwon province | Hyun Ok Yang |
| 9 | *E. hamiltonianus* | IM190603-7 | Nae-myeon, Hongcheon-gun, Gangwon province | Hyun Ok Yang |
| 10 | *E. hamiltonianus* | IM190603-8 | Nae-myeon, Hongcheon-gun, Gangwon province | Hyun Ok Yang |
| 11 | *E. hamiltonianus* | IM190603-9 | Nae-myeon, Hongcheon-gun, Gangwon province | Hyun Ok Yang |
| 12 | *E. hamiltonianus*^a^ | IM190717-2 | Aewol-eup, Jeju-si, Jeju island | Hyun Ok Yang |
| 13 | *E. hamiltonianus* | IM190701-2 | Aewol-eup, Jeju-si, Jeju island | Hyun Ok Yang |
| 14 | *E. hamiltonianus* | IM190701-3 | Aewol-eup, Jeju-si, Jeju island | Hyun Ok Yang |
| 15 | *E. hamiltonianus* | IM190629-1 | Aewol-eup, Jeju-si, Jeju island | Hyun Ok Yang |
| 16 | *E. hamiltonianus* | IM190629-2 | Aewol-eup, Jeju-si, Jeju island | Hyun Ok Yang |
| 17 | *E. hamiltonianus* | IM200511-1 | Daegwallyeong-myeon, Pyeongchang-gun, Gangwon province | Jung wha Kang |
| 18 | *E. hamiltonianus* | IM200511-2 | Daegwallyeong-myeon, Pyeongchang-gun, Gangwon province | Jung wha Kang |
| 19 | *E. hamiltonianus* | IM200511-3 | Hantaek Botanical Garden, Baegam-myeon, Cheoin-gu, Yongin-si, Gyeonggi province | Jung wha Kang |
| 20 | *E. hamiltonianus* | IM200511-4 | Hantaek Botanical Garden, Baegam-myeon, Cheoin-gu, Yongin-si, Gyeonggi province | Jung wha Kang |
| 21 | *E. hamiltonianus* | IM200511-5 | Deoksan-myeon, Jecheon-si, North Chungcheong province | Jung wha Kang |
| 22 | *E. hamiltonianus* | IM200511-6 | Deoksan-myeon, Jecheon-si, North Chungcheong province | Jung wha Kang |
| 23 | *E. hamiltonianus* | IM200511-7 | Songnisan-myeon, Boeun-gun, North Chungcheong province | Jung wha Kang |
| 24 | *E. hamiltonianus* | HT2019-0342 | Dunnae-myeon, Hoengseong-gun, Gangwon province | Jung wha Kang |
| 25 | *E. hamiltonianus* ‘Rising Sun’ | HT2016-0153 | Purchased from Lagg Boskoop 11, 2773 GV Boskoo | Ruper Spaargaren |
| 26 | *E. hamiltonianus* ‘Snow’^a^ | HT2019-0234^b^ | Purchased from Lagg Boskoop 11, 2773 GV Boskoo | Ruper Spaargaren |
| 27 | *E. hamiltonianus* ‘Snow’ | HT2019-0234^b^ | Purchased from Lagg Boskoop 11, 2773 GV Boskoo | Ruper Spaargaren |
| 28 | *E. hamiltonianus* ‘Popcorn’ | HT2016-0152 | Purchased from Lagg Boskoop 11, 2773 GV Boskoo | Ruper Spaargaren |
| 29 | *E. hamiltonianus* ‘Koi Boy’ | HT2016-0151 | Purchased from Lagg Boskoop 11, 2773 GV Boskoo | Ruper Spaargaren |
| 30 | *E. hamiltonianus* | HT2019-0235^c^ | Purchased from Lagg Boskoop 11, 2773 GV Boskoo | Ruper Spaargaren |
| 31 | *E. hamiltonianus* | HT2019-0235^c^ | Purchased from Lagg Boskoop 11, 2773 GV Boskoo | Ruper Spaargaren |
| 32 | *E. europaeus*^a^ | HT2019-0108 | Sannae Botanical Garden, Byeongcheon-myeon, Dongnam-gu, Cheonan, South Chungcheong province | Jung wha Kang |
| 33 | *E. japonicus*^a^ | HT2020-0147 | Geomun-ri, Samsan-myeon, Yeosu, South Jeolla province | Jung wha Kang |

^a^sequenced accessions in Table 2. ^b^ and ^c^ shared voucher numbers, but independent individuals. ^d^vouchers with IM and HT were deposited in Seoul National University and Hantaek Botanical Garden Herbarium, respectively.
