# Supplementary material for: Analysis of the complete plastomes and nuclear ribosomal DNAs from Euonymus hamiltonianus and its relatives sheds light on their diversity and evolution
Source: PLoS One. 2022 Oct 5;17(10):e0275590. doi: 10.1371/journal.pone.0275590 (PMC9534445; doi:10.1371/journal.pone.0275590)
Supplement: S1 Raw images — (PDF) [file pone.0275590.s017.pdf]

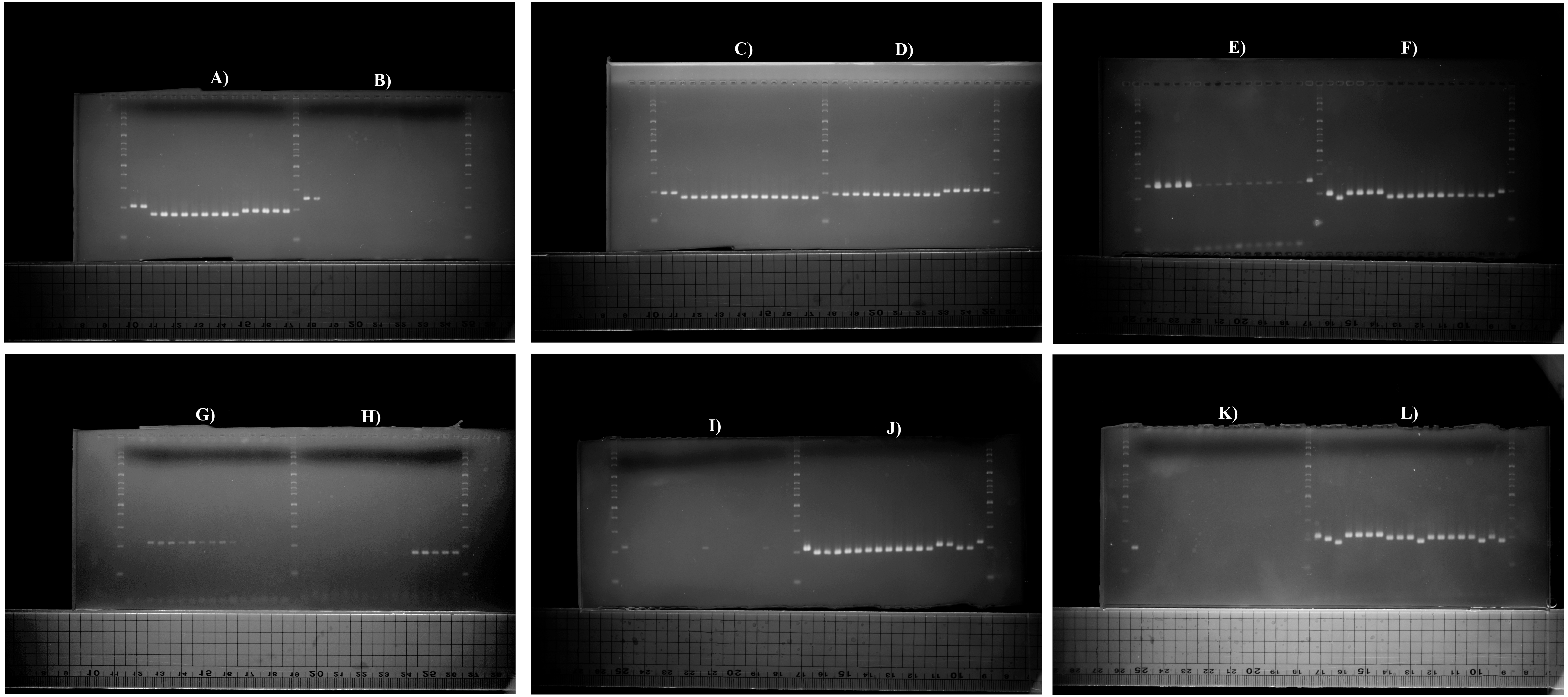

Raw images for Fig 6 and S4-9 Fig.
B and E correspond to S4 Fig. G and I correspond to S5 Fig.
H and K correspond to S6 Fig. C and F correspond to S7 Fig.
D and J correspond to S8 Fig. A and L correspond to S9 Fig.
E, I, K, F, J, and L correspond to Fig 6.
